# Predicted limited redistribution of T cells to secondary lymphoid tissue correlates with increased risk of haematological malignancies in asplenic patients

**DOI:** 10.1038/s41598-021-95225-x

**Published:** 2021-08-12

**Authors:** Aleksandra E. Kmieciak, Liam V. Brown, Mark C. Coles, Jonathan Wagg, Alex Phipps, Eamonn A. Gaffney

**Affiliations:** 1 Wolfson Centre for Mathematical Biology, Mathematical Institute, University of Oxford, Andrew Wiles Building, Woodstock Rd, Oxford, OX2 6GG UK; 2 Kennedy Institute of Rheumatology, University of Oxford, Roosevelt Dr, Headington, Oxford, OX3 7FY UK; 3 AC Immune, EPFL Innovation Park, 1015 Lausanne, Switzerland; 4 Pharmaceutical Sciences-Clinical Pharmacology Roche Innovation Centre Welwyn 6 Falcon Way, Shire Park, Welwyn Garden City, AL7 1TW UK; 5 Present Address: Clinical Pharmacology and Quantitative Pharmacology-Oncoloy, AstraZeneca, Cambridge, CB2 0AA UK

**Keywords:** Cancer, Computational models, Lymphocytes, Lymphoid tissues, Systems biology

## Abstract

The spleen, a secondary lymphoid tissue (SLT), has an important role in generation of adaptive immune responses. Although splenectomy remains a common procedure, recent studies reported poor prognosis and increased risk of haematological malignancies in asplenic patients. The high baseline trafficking of T lymphocytes to splenic tissue suggests splenectomy may lead to loss of blood-borne malignant immunosurveillance that is not compensated for by the remaining SLT. To date, no quantitative analysis of the impact of splenectomy on the human T cell trafficking dynamics and tissue localisation has been reported. We developed a quantitative computational model that describes organ distribution and trafficking of human lymphocytes to explore the likely impact of splenectomy on immune cell distributions. In silico splenectomy resulted in an average reduction of T cell numbers in SLT by 35% (95%CI 0.12–0.97) and a comparatively lower, 9% (95%CI 0.17–1.43), mean decrease of T cell concentration in SLT. These results suggest that the surveillance capacity of the remaining SLT insufficiently compensates for the absence of the spleen. This may, in part, explain haematological malignancy risk in asplenic patients and raises the question of whether splenectomy has a clinically meaningful impact on patient responses to immunotherapy.

## Introduction

The aetiology of cancer is complex and heterogeneous, with considerable variability within and between patients. It is acknowledged that immune surveillance and subsequent response have a pivotal role in the mechanism of cancer defence and progression. Cancer antigen, resulting from the destruction of cancer cells within the tumour microenvironment, are collected by antigen-presenting cells (APCs), that have recognised stress signals. APCs carrying cancer antigen then migrate to secondary lymphoid tissue (SLT), where subsequent T cell activation occurs when a naïve immune T cell encounters an APC that presents antigen specifically recognised by this T cell’s receptor (so-called cognate antigen). Such T cell activation events initiate adaptive immune responses, which drive the identification and selective clearance of abnormal human cells, further facilitating tumour infiltration, killing of cancer cells and antigen release, thus, generating the tumour-immunity cycle.

From a clinical perspective, T cell activation is a pivotal event, as it is believed to be a major driver of efficacy across a range of immunotherapeutic modalities that have emerged as an important advance in cancer treatment^1^. Several strategies have so far yielded promising clinical responses, including CAR T cell therapy and T cell bispecifics being particularly successful in haematological malignancies. Frequent contact of immune and malignant cells in the bloodstream, coupled with malignant cells sharing their cellular origins with those of the immune system, may make haematological malignancies well-suited as targets of immunotherapeutic modalities^2^.

The explanation of immunosurveillance in the development of immune therapeutics and vaccines requires an understanding of both T cell activation and the trafficking of T cells. Initial activation events occur in the SLTs, such as lymph nodes and the spleen, anatomical sites that inter-alia serve as preferential homing sites for naïve T cells. The continuous recirculation of naïve T cells between the circulatory and lymphatic systems provides an efficient mechanism for naïve T cells to encounter APCs presenting cognate antigen^3,4^. A naïve T cell becomes activated when it encounters its cognate antigen expressed in the context of a major histocompatibility complex (MHC) in SLT. As a result, the T cell proliferates rapidly and acquires effector function after a few days^5^. If a cognate antigen is not encountered, the cell leaves the SLT and returns to the blood^6^. In contrast to effector T cells, which have a rapid turnover rate, naïve T cells may recirculate between blood and lymph compartments for prolonged time periods, lasting months in rodents and years in humans^7^. The continuous recirculation of T cells thus facilitates the detection and elimination of any invading pathogens, abnormal human cells or foreign particles^3^.

The process of lymphocyte recirculation is not random but rather governed by selective mechanisms based on lymphocyte-endothelial cell interactions^4^ that occur whilst these cells are also subject to the hydrodynamic shear stress exerted by flowing blood^5^. Although the lymphocyte-endothelial cell interactions are mediated by several distinct molecular pathways, these interactions are tissue- and lymphocyte-specific, i.e., usually only a single combination of interaction molecules enables efficient migration of a lymphocyte population to a specific target tissue^5^. The migration of a given T cell subset is therefore supported in a specific tissue, while endothelial cells everywhere else permit little or no lymphocyte binding, preventing subsequent extravasation^8^.

Although the spleen comprises a major component of SLT, studies of mechanisms of lymphocyte trafficking and activation have focused predominantly on lymph nodes (LNs). LNs, which are widely present throughout the human body and often arranged in chains, drain the interstitial tissues of cells, proteins and surplus tissue fluid (collectively referred to as lymph) via afferent lymphatic vessels. Components of lymph that may present or contain antigen are removed within the LN, whilst other components, such as excess fluid, return to the central circulation via efferent lymphatic vessels^9^. Unlike LNs, however, the spleen lacks afferent lymphatic vessels, thus all cells and antigens enter the organ directly via the blood^6^. The distinct structural differences and the high blood perfusion through the spleen^10^ highlight the spleen’s immunological role in a wider context—the spleen not only serves a significant role as an anatomical site for lymphocyte activation due to its higher cellular capacity but also performs critical haematological functions, such as clearing the blood of bacteria and aged, antibody-coated or damaged cells^11^. Additionally, the spleen has a key role in regulating iron levels, removal of damaged red blood cells and storage of platelets^11^. Furthermore, given the high blood flow and, consequently, relatively high influx of cells into the spleen, the organ is a crucial site of early exposure to blood-borne pathogens, malignant cells and intravenously administered therapies.

Splenectomy is a relatively common procedure with 22,000 procedures performed annually for all causes in the USA^12^. As the spleen, being a major component of SLT, is an important site of immune response generation, it is possible that splenectomy may compromise the immune system’s ability to both prevent and subdue cancer. Two recent cohort studies^13,14^ investigated the association between splenectomy and malignancy. Kristinsson et al. assessed incidence and mortality due to potential complications of splenectomy, including cancer, among 8149 splenectomised American veterans with a follow-up to 27 years^13^. Sun et al. conducted a nation-wide cohort study of 2604 splenectomised Taiwanese patients, with a follow-up of up to 11 years^14^. These studies reported analyses based on Standardised Incidence and Mortality Ratios, SIR and SMR, respectively. The ratios estimate the occurrence of an event in a given population relative to a larger comparator population, used as a reference group, thereby giving a statistical measure of what the expected incidence of an analysed condition (SIR) and the expected number of deaths due to a condition (SMR) would be in a population of interest, given a matched cohort^15^. It is worth noting that, while both SIR and SMR are adjusted for factors differentiating the studied and reference groups (age, gender, pre-existing health conditions, etc.), SMR does not account for a changing standard of care and hence necessitates a more careful interpretation.

**Figure 1:**
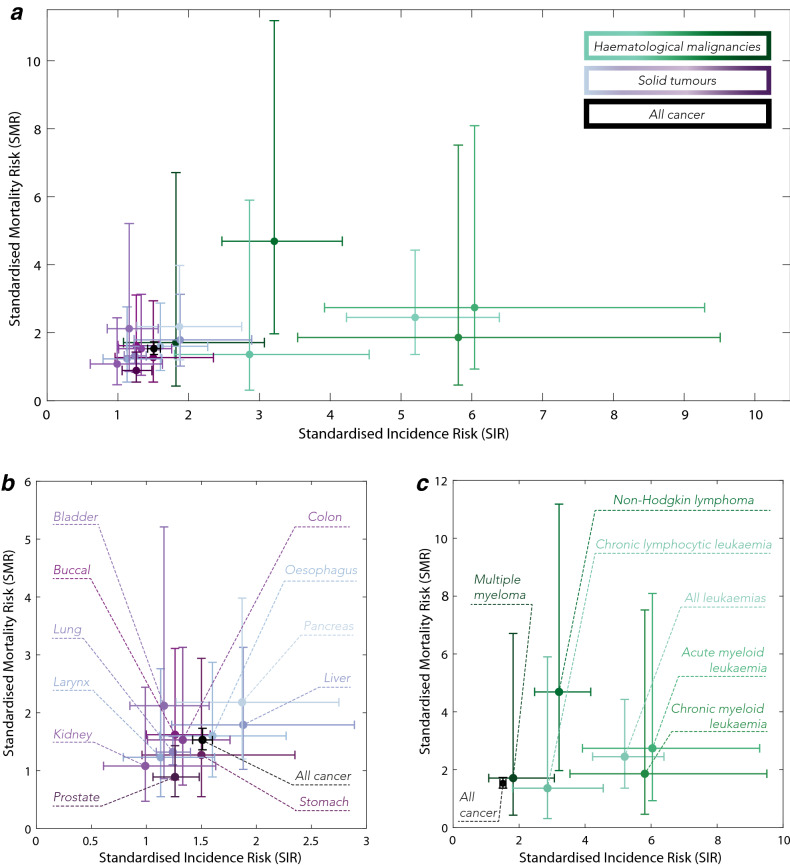
Standardised Incidence Ratio (SIR) and Standardised Mortality Ratio (SMR) due to selected malignancies following splenectomy as reported by Kristinsson et al.^13^. Reported malignancies were plotted on one graph (**a**) to highlight the visible distinction between solid tumours (**b**) and haematological malignancies (**c**). Cross-lines represent the reported 95% confidence intervals (95%CI). Note the difference in scale.

Kristinsson et al. and Sun et al. studies reported an increased overall incidence ratio for any malignancy following splenectomy: 1.51 (95%CI 1.42–1.46)^13^ and 2.06 (95%CI 1.81–2.33)^14^, respectively. Both analyses also revealed significantly higher incidence ratios with splenectomy for the haematological malignancies, not only compared to an overall cancer incidence but also compared to solid tumour incidence (see Fig. 1 for visualisation of the reported data). SIRs were raised across all haematological malignancies in asplenic versus spleen intact populations in both studies, ranging from 1.82 (95%CI 1.08–3.07) for multiple myeloma^13^ up to 6.04 (95%CI 3.92–9.29) for acute myeloid leukaemia^13^. Across haematological malignancies, all leukaemias and Non-Hodgkin lymphoma were associated with a statistically significant increase in SMR: 2.45 (1.36–4.43) and 4.69 (1.97–11.18), respectively^13^.

The data highlights the importance of the spleen in immunosurveillance, as the organ plays an essential role, not just for protection against blood-borne bacterial infections, but any kind of abnormal cell carried in the blood. While the process of tumour antigen presentation to T cells is almost completely restricted to the tumour-draining lymph nodes are^16,17^, the spleen might be a more efficient anatomical site to mobilise an immune response against blood-borne malignant cells, and potentially associated treatments. As such, the data raises a question of whether splenectomised patients should undergo cancer immunotherapies, given the associated severe side effects and, effectively, an already impaired immune system due to splenectomy. The splenectomy covariate has so far not been systematically explored for its impact on treatment outcome. At the time of writing, and in the context of asplenic patients, there are no published clinical trial results for two recently approved CAR T cell therapies, Kymriah (Tisagenlecleucel) and Yescarta (Axicabtagene ciloleucel), and a bispecific T cell engager Blincyto (Blinatumomab). Further research is needed to provide input into considerations of the appropriate treatment for splenectomised patients with haematological malignancies. Model-based approaches could facilitate elucidating the impact of splenectomy on T cell trafficking, in particular, the prospect of induced changes in trafficking through lymph nodes to investigate whether a compensation mechanism is even physiologically feasible.

A modelling approach particularly suited for this purpose is Physiologically Based Pharmacokinetic (PBPK) modelling, which has been established since the early 1980s^18^ and is frequently used to predict a systemic and tissue exposure of a drug^19^. A PBPK framework comprises of compartments corresponding directly and faithfully to the organs and tissues of the body, and connected by the cardiovascular system. Such descriptions of physiological systems can be then solved computationally, providing insight into the underlying biological mechanisms^19^. Although usually applied to drugs and antibodies, the methodology has also been utilised to describe the distribution and trafficking dynamics of cells and cellular therapies^20–26^.

In this study, we refined a previously constructed PBPK framework of T cell trafficking^27^ to understand the likely distributions of T cells across different SLT compartments in the context of splenectomy. The unique advantages of PBPK modelling, such as anatomically-based model design and incorporation of measurable quantities (*e.g.*, organ volumes, blood flow rates) as model parameters, have been utilised here to determine predictions of the immune cell biodistribution in the absence of the spleen. Since, to the best of our knowledge, immune cell trafficking in relation to splenectomy has only been modelled in mice^22^, we have assessed how in silico splenectomy affects T cell distribution in human. In particular, we have investigated the effect of splenectomy on the number (localisation) and concentration of T cells in lymph nodes (exposure), examining the clinically relevant question of to what extent the remaining SLT post-splenectomy may have the capacity to compensate for the lost immunosurveillance, even before differential function between the spleen and lymph nodes is considered. Furthermore, we aim to highlight the impact of splenectomy on cell trafficking and, in particular, changes in the bioavailability of an administration of T cells in the SLT, which is pertinent to cell based immunotherapy.

## Methods

### Model summary

**Figure 2:**
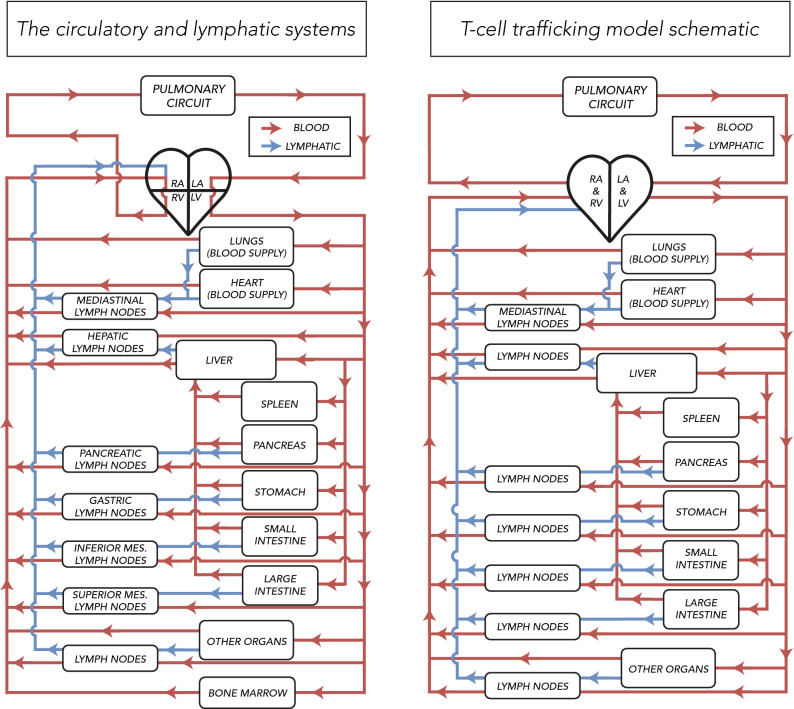
Model schematic of the ODE system (right) compared to the simplified schematic of the blood and lymph circulation (left). As a PBPK framework, the T cell trafficking model comprises of anatomical compartments corresponding directly and faithfully to the organs and tissues of the body, connected by the cardiovascular and lymphatic system. Such a description of anatomy is used to predict a systemic and tissue exposure to the circulating T cells. Cells flow from the left atrium and left ventricle of the heart to each organ (red lines), via lymphatics (blue lines) into organ-draining lymph nodes and return back to the right atrium and right ventricle of the heart. Each box represents a vasculature and interstitium compartment. Chambers of the heart: *RA*—right atrium, *RV*—right ventricle, *LA*—left atrium, *LV*—left ventricle. *Other organs* compartments include: adrenals, bladder, brain, adipose tissue, gonads, kidneys, skeletal muscles, skeleton, skin, thyroid. Note that the skeleton compartment in the model includes the bone marrow and bones and is parameterised accordingly (see Table 2).

**Figure 3:**
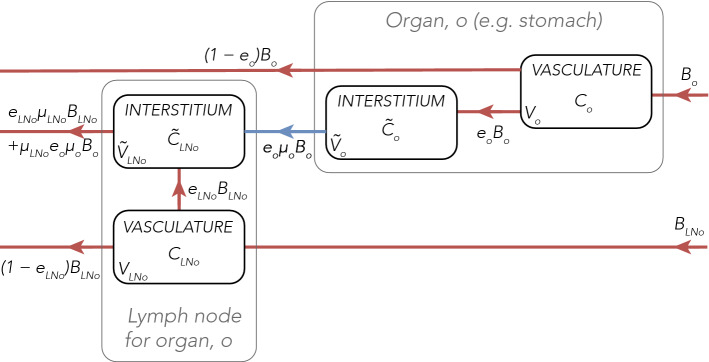
Organ, *o*, and draining lymph nodes for organ, *o*, are represented in the model as separate vascular and interstitial compartments. T cells flow from the cardiac output compartment (previously defined as left atrium, left ventricle, aorta and large arteries) to each organ, *o*, with a blood flow $B_o$, from which a proportion, $e_o B_o$, enters the interstitial space per unit time. A proportion of T cells, $e_o \mu _o B_o$, flows via lymphatics from the interstitial sub-compartment of organ, *o*, into the draining lymph nodes per unit time. T cells also enter organ, *o*, -draining lymph nodes through the lymph node blood supply, denoted $B_{\text {LN}_o}$. From the lymph node vasculature compartment, a proportion of T cells extravasates into the lymph node interstitial compartment (at a rate $e_{\text {LN}_o} B_{\text {LN}_o}$). T cells drained to organ, *o*, -draining lymph nodes are returned to the systemic circulation (at a rate $e_{\text {LN}_o} \mu _{\text {LN}_o} B_{\text {LN}_o} + \mu _{\text {LN}_o} e_o \mu _o B_o$). T cells that have not extravasated to the interstitium of organ, *o*, and organ, *o*, -draining lymph nodes are, similarly, returned to the systemic circulation (at rates $(1-e_o)B_o$ and $(1-e_{\text {LN}_o})B_{\text {LN}_o}$, respectively). Different suffixes, such as *LA&LV* (combined left atrium, left ventricle, and large arteries), *RA&RV* (combined right atrium, right ventricle, and large veins), *PC* (pulmonary circuit), *med* (mediastinal lymph nodes) are also used.

A system of ordinary differential equations (ODEs) has been constructed to represent the exchange of any population of recirculating cells between the heart and organs, with an underlying context in this study of T cell trafficking and T cell immunotherapies. “Model overview” and “Lymph nodes—whole-body distribution” sections provide an outline of the model’s structure and its mathematical description. The full model is represented by Fig. 2 (right) with definitions and units of parameters and variables in the model shown in Table 1. Individual organs are modelled as shown in Fig. 3. This corresponds to Eqs. (1) and (2) for organs other than the liver, spleen, mesentery and lymph nodes, where the extra complications of this anatomy, as depicted in Fig. 2, is represented by Equations (S.3)–(S.9) of the Supplementary Material. The parameterisation of the model is discussed in detail below, with the resulting parameter ranges summarised in Table  2.

**Table 1:** Definitions and units of parameters and variables in the model described in “Model overview” section.

Symbol	Definition	Unit
$C_o$	T cell vasculature concentration in organ, *o*	Cells/L
${\tilde{C}}_o$	T cell interstitial volume concentration in organ, *o*	Cells/L
$C_{LN_o}$	T cell vasculature concentration in lymph node for organ, *o*	Cells/L
${\tilde{C}}_{LN_o}$	T cell interstitial volume concentration in lymph node for organ, *o*	Cells/L
$N_o$	Number of T cells in the vasculature of organ, *o*	–
${\tilde{N}}_o$	Number of T cells in the interstitial space of organ, *o*	–
$N_{LN_o}$	Number of T cells in the vasculature of lymph node for organ, *o*	–
${\tilde{N}}_{LN_o}$	Number of T cells in the interstitial space of lymph node for organ, *o*	–
$B_o$	Blood flow (volume of blood per unit time) in organ, *o*	L/min
$B_{LN_o}$	Blood flow (volume of blood per unit time) in lymph node for organ, *o*	L/min
$V_o$	Blood volume in organ, *o*	L
$V_{LN_o}$	Blood volume in lymph node for organ, *o*	L
${\tilde{V}}_o$	Total volume of organ, *o*	L
${\tilde{V}}_{LN_o}$	Total volume of lymph node for organ, *o*	L
$e_o$	Fraction of cells extravasating into the interstitial space of organ, *o*	–
$e_{LN_o}$	Fraction of cells extravasating into the interstitial space of lymph node for organ, *o*	–
$\mu _o$	Fraction of cells leaving the interstitial space of organ, *o*	–
$\mu _{LN_o}$	Fraction of cells leaving the interstitial space of lymph node for organ, *o*	–

Different suffixes, such as *LA&LV* (left atrium, left ventricle, and large arteries), *RA&RV* (right atrium, right ventricle, and large veins), *PC* (pulmonary circuit), *med* (mediastinal lymph nodes) are also used. See Fig. 3 for a schematic of an exemplar organ and the associated parameters.

### Model overview

The whole-body physiologically-based model treats the major human organs as compartments connected in an anatomical manner by the systemic and lymphatic circulation. Figure 2 shows a diagram of the circulatory and lymphatic systems (left) and contrasts it with a simplified schematic of the T cell trafficking framework (right). The model contains 20 organs, shown in Fig. 2, each with a vascular and interstitial compartment, and two additional heart chambers compartments. The organs in the model include: adrenals, bladder, brain, fat, gonads, blood supply to the heart muscle, contents of the heart chambers, kidneys, large intestine, liver, lungs, lymph nodes, muscles, pancreas, pulmonary circuit, small intestines, skeleton (bones and bone marrow), skin, spleen, stomach and thyroid. The blood supply to the heart muscle (coronary circulation) is represented as a separate vascular and interstitial compartment, in addition to a heart chamber compartments due to a distinct anatomy of blood circulation (see Fig. 2 for further details, including the organs in the model). Similarly, pulmonary circulation and lung tissue blood supply are treated as separate compartments. To provide a more physiologically-realistic description, the original model developed by Brown et al.^27^ was extended to represent lymph node compartments as multiple compartments distributed across the model. In particular, a separate group of lymph nodes, treated as a single ‘lumped’ compartment, drains each organ’s interstitium, as depicted in Fig. 2. The exceptions are the blood supply to the lungs and heart, where a single lymph node compartment drains both organs (mediastinal lymph nodes), the spleen, which lacks draining lymphatic vessels, and the pulmonary circuit, where the interstitial sub-compartment represents the state of cell entrapment in the pulmonary vasculature^28,29^.

The independent variables are the number of T cells in each of the compartments as a function of time. The number of T cells in a given compartment of an organ, *o*, is denoted by $N_o$, with a rate of change driven by the difference in the rate of flow of these cells into and out of the compartment. The flow of T cells is proportional to the blood flow and cell concentration^30^. For instance, the rate of change in the number of T cells in the vasculature of an organ, *o*, is given by:

1 $$\frac{{d{N_o}}}{{dt}} = {V_o}\frac{{d{C_o}}}{{dt}} = {B_o}\left( {{C_{{\text{LA}}\&{\text{LV}}}} - {C_o}} \right),$$

where $V_o$ is the blood volume of the organ, *o*, $B_o$ is the blood flow of the organ, *o*, while $C_{{{\text{LA}}{\&}{\text{LV}}}}$ is the concentration of T cells within the left atrium, left ventricle, aorta and large arteries between organs and the heart.

To reflect the fact that a proportion of cells in the vasculature extravasates into the organ’s interstitium, a fraction of cells in the vasculature of the organ, *o*, denoted $e_o$, flows from that organ’s vasculature sub-compartment into the interstitial sub-compartment (see Fig. 3). The extravasation factor, $e_o$, is assumed to be constant for a given organ, *o*. While the process of extravasation of immune cells can be modified by integrins and other molecular factors^31^, it is assumed that the extravasation factor is constant on the timescale of the simulation of interest. Hence, the rate of extravasation is proportional to the flow of T cells out of the vasculature sub-compartment: $e_oB_oC_o$.

From the interstitium of organ, *o*, T cells flow via lymphatics into its draining group of lymph nodes (see Fig. 3). A fraction of T cells, $\mu _o$, flows from the interstitial sub-compartment of organ, *o*, into the draining lymph nodes. Similarly to the extravasation factor ($e_o$), the return factor, $\mu _o$, is assumed to be constant for a given organ, *o*, and the T cell return rate is proportional to the flow of cells out of interstitium: $e_o\mu _oB_o{\tilde{C}}_o$, where ${\tilde{C}}_o$ is the interstitial concentration of T cells. As a result, the number of T cells in the interstitial compartments, ${\tilde{N}}_o$, changes according to the following equation:

2 $$\begin{aligned} \frac{d{\tilde{N}}_o}{dt} = {\tilde{V}}_o\frac{d{\tilde{C}}_o}{dt} = e_oB_o \left( C_o - \mu _o{\tilde{C}}_o \right) , \end{aligned}$$

where ${\tilde{V}}_o$ is the volume of the organ.

Equations (1) and (2) apply to all organs except the liver and lymph nodes, the equations for which can be found in Supplementary Material. Equations for liver need to be modified to capture the specific anatomy of hepatic blood and lymph circulation. The liver receives blood exiting the mesenteric organs: the stomach, spleen, pancreas, small and large intestines. Note that since the spleen does not have any afferent lymphatics, the liver receives both the T cells that have extravasated into the spleen and those that have not.

Each group of organ-draining lymph nodes is represented by a vasculature sub-compartment (the blood supply to the lymph node) and the interstitial sub-compartment, where T cells from the interstitium of organ, *o*, drain to via lymphatics (as shown in Fig. 3). The change in the number of T cells in the lymph nodes’ blood supply compartment is analogous to Eq. (1), while the change of T cells in the interstitial lymph node compartment accounts for the contribution from the lymph nodes’ vasculature and, additionally, the flow of interstitium drained cells through organ, *o* (Fig. 3). Due to the specific anatomy of hepatic blood circulation and, therefore, different flow of T cells from the liver, the equation for the change in the number of T cells in liver-draining lymph nodes is modified accordingly (see Supplementary Material for explicit forms of the equations). Finally, T cells that have not extravasated from the organ’s vasculature, combined with the lymph node outflow, return to the heart (right atrium and right ventricle), the pulmonary circuit, and then back to the heart chambers (left atrium and left ventricle). While T cells undergo the processes of proliferation in the bone marrow and depletion in various tissues, the rates at which T cells are produced and depleted are assumed to be zero, given the timescale of the simulation of interest. The full system of ODEs describing the whole-body dynamics of T cell trafficking can be found in Supplementary Material.

### Lymph nodes—whole body distribution

To describe the distribution of lymph nodes within the model, certain assumptions have been made due to the lack of detailed literature on lymphatic drainage pathways. It was assumed that lymph nodes each drain a constant volume of tissue. In such a case, the number of lymph nodes draining a particular organ increases with the organ’s volume, and lymph nodes were, therefore, partitioned by organ volume. The volume of the group of lymph nodes draining an organ, *o*, ${\tilde{V}}_{\text {LN}_o}$, the blood flow, $B_{\text {LN}_o}$, and the blood volume of that lymph node group, $V_{\text {LN}_o}$, were assumed as fractions of the total values of those parameters (see Supplementary Material) using the partitioning factor:

3 $$\begin{array}{l}
{{\tilde V}_{{\text{L}}{{\text{N}}_o}}} = \frac{{{{\tilde V}_o}}}{{\sum\limits_{i \notin \left\{ {{\text{spleen}},{\text{PC}},{\text{RA}}\& {\text{RV}},{\text{LA}}\& {\text{LV}}} \right\}} {{{\tilde V}_i}} }}{{\tilde V}_{{\text{L}}{{\text{N}}_{{\text{TOT}}}}}}\\
{B_{{\text{L}}{{\text{N}}_o}}} = \frac{{{{\tilde V}_o}}}{{\sum\limits_{i \notin \left\{ {{\text{spleen}},{\text{PC}},{\text{RA}}\& {\text{RV}},{\text{LA}}\& {\text{LV}}} \right\}} {{{\tilde V}_i}} }}{B_{{\text{L}}{{\text{N}}_{{\text{TOT}}}}}}\\
{V_{{\text{L}}{{\text{N}}_o}}} = \frac{{{{\tilde V}_o}}}{{\sum\limits_{i \notin \left\{ {{\text{spleen}},{\text{PC}},{\text{RA}}\& {\text{RV}},{\text{LA}}\& {\text{LV}}} \right\}} {{{\tilde V}_i}} }}{V_{{\text{L}}{{\text{N}}_{{\text{TOT}}}}}},
\end{array}$$

where ${\tilde{V}}_o$ is the volume of the drained organ (or sum of organ volumes, for the mediastinal lymph node compartment), and the index *i* runs over all organs drained by lymph nodes (so that the sum represents the total volume of all organs drained via lymphatics), ${\tilde{V}}_{\text {LN}_{\text {TOT}}}$, $B_{\text {LN}_{\text {TOT}}}$ and $V_{\text {LN}_{\text {TOT}}}$ are the total volume of lymph nodes, the total blood flow to lymph nodes and the total blood volume in lymph nodes, respectively.

### Parameterisation

Extravasation and return parameters for all organs, $e_o$ and $\mu _o$, and organ-draining lymph nodes, $e_{\text {LN}_o}$ and $\mu _{\text {LN}_o}$, were defined to vary uniformly between 0 and 1. Blood flows, blood and organ volumes in the model were parameterised based on the literature review of relevant physiological reference data carried out by Brown et al.^27^. To avoid using data from different studies, the most complete set of reference data was chosen for baseline parameter values, namely the International Commission on Radiological Protection (ICRP) compilations^32,33^. The ICRP publications have taken into account age- and gender-related differences in the anatomical and physiological characteristics of reference individuals and have been valued as a standardised set of human data for medical purposes. Since anatomical values vary greatly between and within individuals, the variability between sources compiled by Brown et al.^27^ was used as a proxy for anatomical variability. The reported parameter variability was extended by identifying parameters associated with high temporal and inter-individual variability in human^32^, i.e., the muscle^34,35^ and fat^36,37^ volumes, the blood flows through organs whose blood flows change as a result of exercise (muscle^38^, lungs^39^ and pulmonary circuit^39^), meal (mesentery^40^) or temperature change (skin^41^). For each parameter, a wider range was chosen from all reported values and, when only one parameter value was available, we assumed a uniform variability of 25% from the baseline value. A summary of the resulting parameter ranges can be found in Table 2.

**Table 2:** Human anatomical parameter values and variability ranges used for the model parameterisation.

Organs	Blood flow [L/min]		Blood volume [L]		Organ volume [L]	
	Baseline	Variability ranges	Baseline	Variability ranges	Baseline	Variability ranges
Adrenals	0.019^32^	0.011–0.022^27^	0.032^32^	0.001–0.007^27^	0.017^32^	0.014–0.017^27^
Bladder	0.004^32^	0.003–0.005^27^	0.001^32^	0.0008–0.0013^27^	0.048^32^	0.036–0.06^27^
Brain	0.78^32^	0.477–1.001^27^	0.064^32^	0.019–0.593^27^	1.381^32^	1.381–1.45^27^
Fat	0.325^32^	0.194–0.484^27^	0.265^32^	0.094–0.906^27^	19.791^32^	2.249–31.5^36,37^
Gonads	0.0033^32^	0.0016–0.0041^27^	0.0021^32^	0.0048–0.0026^27^	0.017^32^	0.017–0.036^27^
Heart (blood supply)	0.26^32^	0.139–0.326^27^	0.053^32^	0.0085–0.734^27^	0.314^32^	0.266–0.341^27^
Kidneys	1.235^32^	0.704–1.464^27^	0.106^32^	0.012–0.243^27^	0.295^32^	0.295–0.332^27^
Large Intestine	0.26^32^	0.198–0.554^27,40^	0.53^32^	0.006–0.262^27^	0.356^32^	0.356–0.548^27^
Liver	0.423^32^	0.049–0.113^27,40^	0.117^32^	0.069–1.19^27^	1.714^32^	1.687–2.143^27^
Lungs	0.163^32^	0.096–1^27,39^	0.663^32^	0.036–1.487^27^	1.142^32^	0.519–1.169^27^
Lymph nodes	0.11^32^	0.087–0.241^27^	0.011^32^	0.002–0.038^27^	0.709^32^	0.274–0.709^27^
Skeletal muscles	1.105^32^	0.544–35^27,38^	0.742^32^	0.098–10.831^27^	27.619^32^	20–45.5^34,35^
Pancreas	0.065^32^	0.053–0.115^27^	0.032^32^	0.004–0.071^27^	0.133^32^	0.084–0.133^27^
Small intestine	0.65^32^	0.344–1.385^27,40^	0.201^32^	0.004–0.533^27^	0.625^32^	0.385–1.652^27^
Skeleton (bone and bone marrow)	0.325^32^	0.159–0.333^27^	0.371^32^	0.102–1.77^27^	7.726^32^	7.164–30.078^27^
Skin	0.325^32^	0.199–8^27,41^	0.03^32^	0.03–0.12^27^	3.562^32^	3.408–7.77^27^
Spleen	0.195^32^	0.091–0.199^27^	0.074^32^	0.011–0.167^27^	0.144^32^	0.144–0.221^27^
Stomach	0.065^32^	0.03–0.138^27,40^	0.053^32^	0.008–0.119^27^	0.144^32^	0.143–0.154^27^
Thyroid	0.098^32^	0.033–0.121^27^	0.003^32^	0.0011–0.0071^27^	0.019^32^	0.019–0.02^27^
Pulmonary circuit	–		0.557^32^	0.417–0.696	0.557^32^	0.417–0.696
Right atrium and ventricle	–		1.193^32^	0.894–1.491	–	
Left atrium and ventricle	–		0.557^32^	0.417–0.696	–	

The baseline parameter values come from the International Commission on Radiological Protection (ICRP) compilations^32,33^. The variability ranges were based on the parameter value selection made by Brown et al.^27^ and additional selected literature sources^34–41^. Note that liver row does not include the portal vein, and the pulmonary circuit is listed separately from the lung blood supply parameters. Also note that the parameterisation for the heart chambers includes the blood volume of major blood vessels—superior and inferior vena cavae (for the right ventricle and atrium) and aorta (for the left atrium and ventricle), while the skeleton parameterisation corresponds to an amalgam of bone and bone marrow (see Supplementary Material for more details).

### Exemplar simulation

**Figure 4:**
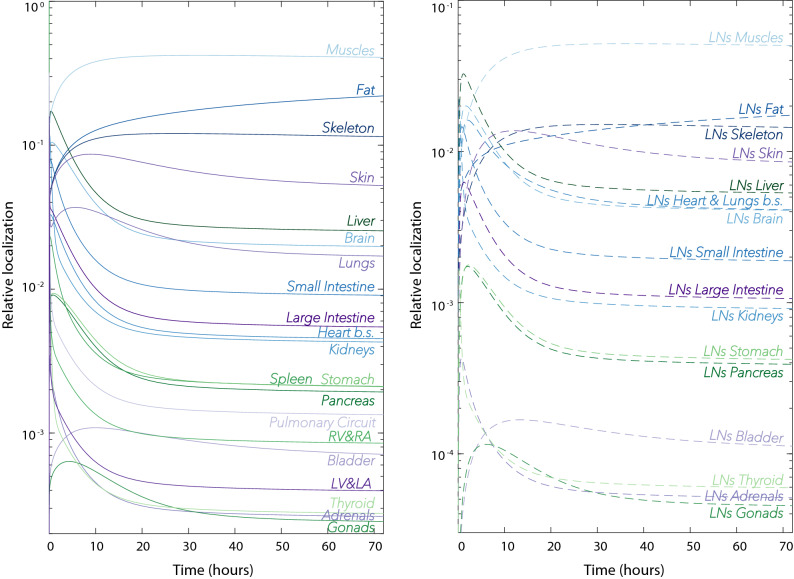
Example results from the ODE system, subject to the initial distribution of T cells administered intravenously (Eq. 4), with baseline parameter values (see Table S.1, Supplementary Material). Each curve represents the sum of the number of T cells in vascular ($N_o$) and interstitial localisations (${\tilde{N}}_o$) expressed as fraction of the total number of administered T cells (Relative localisation). For all organs (left) and all organ-draining groups of lymph nodes (right), extravasation, $e_o$, and return parameters, $\mu _o$, were assumed to be equal to 0.25 and 0.05, respectively. Note that the contents of heart chambers (*RA&RV* and *LA&LV*) and the blood supply to the heart (Heart blood supply) are plotted separately. Similarly, the lung curve does not include the pulmonary circuit.

The ODE system was solved numerically using the Matlab stiff solver ode15s with a variable time step, subject to initial conditions, where T cells of interest are administered intravenously, i.e., to the right atrium, right ventricle and large veins compartment (*RA&RV*):

4 $$\begin{aligned}
{N_{{\text{RA}}\& {\text{RV}}}}\left( 0 \right) & = {V_{{\text{RA}}\& {\text{RV}}}}{C_{{\text{RA}}\& {\text{RV}}}}\left( 0 \right)  = {N_{{\text{TOT}}}},\\
{N_o}\left( 0 \right) & = {V_o}{C_o}\left( 0 \right) = 0,\\
{{\tilde N}_o}\left( 0 \right) & = {{\tilde V}_o}{{\tilde C}_o}\left( 0 \right)  = 0,\\
{N_{{\text{L}}{{\text{N}}_o}}}\left( 0 \right) & = {V_{{\text{L}}{{\text{N}}_o}}}{C_{{\text{L}}{{\text{N}}_o}}}\left( 0 \right)  = 0,\\
{{\tilde N}_{{\text{L}}{{\text{N}}_o}}}\left( 0 \right) & = {{\tilde V}_{{\text{L}}{{\text{N}}_o}}}{{\tilde C}_{{\text{L}}{{\text{N}}_o}}}\left( 0 \right)  = 0,\\\end{aligned}$$

where $N_{\text {TOT}}$ is the total number of T cells of interest in the system. Figure 4 shows an example solution for the ODE system.

### Steady-state model reduction

A reduced framework was derived to identify the determinants governing the response of the system to splenectomy and offer additional structural and biological insight. The ratio of post- to pre-splenectomy cell concentration in any compartment can be derived analytically by considering the ODE system at steady-state. The outline of the derivation is presented here, while the detailed mathematical treatment can be found in Supplementary Material.

Denoting the pre-splenectomy vascular cell concentration in organ, *o*, as $C_o$, and the post splenectomy vascular cell concentration as $C_o^*$, Eq. (1) in steady state implies that $C_o = C_{{{\text{LA}}{\&}{\text{LV}}}}$ pre-splenectomy and $C_o^* = C^*_{{{\text{LA}}{\&}{\text{LV}}}}$ post-splenectomy for all organs with the exception of the liver. We can, therefore, define a dimensionless constant $\kappa$, such that

5 $$  \begin{aligned} \kappa = \frac{C_o^*}{C_o} = \frac{C_{{{\text{LA}}{\&}{\text{LV}}}}  ^*}{C_{{{\text{LA}}{\&}{\text{LV}}}}  }, \end{aligned}$$

the post- to pre-splenectomy vascular cell concentration ratio, equal for all vasculature sub-compartments, except the liver.

Similarly, by considering Eq. (2), the steady-state cell concentration in the interstitium of organ, *o*, (${\tilde{C}}_o$) can be related to the steady-state cell concentration in the corresponding vasculature compartment ($C_o$): $\mu _o{\tilde{C}}_o = C_o$ (pre-splenectomy) and $\mu _o{\tilde{C}}^*_o = C^*_o$ (post-splenectomy). The interstitial cell concentration pre- and post-splenectomy in the organ, *o*, are related by the same constant, $\kappa$, analogously to Eq. (5):

6 $$\begin{aligned} \frac{{\tilde{C}}^*_o}{{\tilde{C}}_o} = \kappa . \end{aligned}$$

Except for liver, the post- to pre-splenectomy cell concentration ratios are therefore equal for all interstitial sub-compartments, including lymph node interstitial sub-compartments (as further detailed in the Supplementary Material).

Due to the distinct ODE structure, the post- to pre-splenectomy cell concentration ratios are expected to differ for the liver and the group of liver-draining lymph nodes. The derivation follows in the same manner, that is, by considering the equations in the steady-state and substituting the above-derived relations, the post- to pre-splenectomy cell concentration ratios can be simplified to

7 $$\begin{aligned} \frac{C^*_{\text {liver}}}{C_{\text {liver}}} = \frac{{\tilde{C}}^*_{\text {liver}}}{{\tilde{C}}_{\text {liver}}} = \frac{\kappa }{\lambda }, \end{aligned}$$

where $\kappa$ was introduced above, $\lambda$ is given via $\lambda = C_{\text {liver}}/C_o$, and

8 $$\begin{aligned} \frac{{\tilde{C}}^*_{\text {LN}_{\text {liver}}}}{{\tilde{C}}_{\text {LN}_{\text {liver}}}} = \frac{\sigma ^*\kappa }{\sigma }, \end{aligned}$$

where $\sigma$ and $\sigma ^*$ are given via $\sigma = \mu _{\text {LN}_{\text {liver}}} {\tilde{C}}_{\text {LN}_{\text {liver}}}/C_o$ and $\sigma ^* = \mu _{\text {LN}_{\text {liver}}} {\tilde{C}}^*_{\text {LN}_{\text {liver}}}/C^*_o$, respectively, and $\lambda$, $\sigma$, $\sigma ^*$ are functions of model parameters (see Supplementary Material for the explicit forms in terms of model parameters).

Finally, an expression for $\kappa$, the post- to pre-splenectomy concentration ratio (in organs other than the liver and liver-draining lymph nodes) is derived from the constraint that the total number of T cells in the system is conserved pre- and post-splenectomy: $N_{\text {TOT}} = N^*_{\text {TOT}}$. Expressing the constraint as the sum of cell numbers across each compartment in the steady state, and substituting the derived expression for the cell concentration pre- and post-splenectomy in the liver and lymph node compartments yields an explicit expression for $\kappa$ (see Supplementary Material).

Given all derived ratios, two quantities of interest were constructed: post- to pre-splenectomy total SLT T cell localisation ratio (the ratio of the steady-state number of T cells in SLT compartments post- to pre-splenectomy, $N^*_{\text {SLT}}/N_{\text {SLT}}$) and post- to pre-splenectomy net SLT T cell concentration ratio (the ratio of the steady-state concentration of T cells in SLT compartments post- to pre-splenectomy, $C^*_{\text {SLT}}/C_{\text {SLT}}$):

9 $$    \begin{aligned} \begin{aligned} \frac{N^*_{\text {SLT}}}{N_{\text {SLT}}} = \kappa \frac{ \sum \limits _{ {\begin{array}{c} o \notin \{ \text {spleen,RA} {\&} \text {RV,} \\ \text {PC,LA} {\&} \text {LV,liver}\} \end{array}} } \left( V_{\text {LN}_{o}} + {\tilde{V}}_{\text {LN}_{o}}\frac{1}{\mu _{\text {LN}_{o}}} \right) + V_{\text {LN}_{\text {liver}}} + {\tilde{V}}_{\text {LN}_{\text {liver}}}\frac{\sigma ^*}{\mu _{\text {LN}_{\text {liver}}}} }{ V_{\text {spleen}} + {\tilde{V}}_{\text {spleen}}\frac{1}{\mu _{\text {spleen}}} + \sum \limits _{ {\begin{array}{c} o \notin \{ \text {spleen,RA} {\&} \text {RV,} \\ \text {PC,LA} {\&} \text {LV,liver}\} \end{array}} } \left( V_{\text {LN}_{o}} + {\tilde{V}}_{\text {LN}_{o}}\frac{1}{\mu _{\text {LN}_{o}}} \right) + V_{\text {LN}_{\text {liver}}} + {\tilde{V}}_{\text {LN}_{\text {liver}}}\frac{\sigma }{\mu _{\text {LN}_{\text {liver}}}}}, \end{aligned} \end{aligned}$$

10 $$    \begin{aligned} \frac{{\bar{C}}^*_{\text {SLT}}}{{\bar{C}}_{\text {SLT}}} = \left. \frac{N^*_{\text {SLT}}}{N_{\text {SLT}}} \Bigg / \frac{ \sum _{ {\begin{array}{c} o \notin \{ \text {spleen,RA} {\&} \text {RV,} \\ \text {PC,LA} {\&} \text {LV}\} \end{array}} } {\tilde{V}}_{\text {LN}_{o}} }{ {\tilde{V}}_{\text {spleen}} + \sum _{ {\begin{array}{l} o \notin \{ \text {spleen,RA} {\&} \text {RV,} \\ \text {PC,LA} {\&} \text {LV}\} \end{array}} } {\tilde{V}}_{\text {LN}_{o}} } \right. . \end{aligned}$$

where the summations run over groups of lymph nodes, and $\kappa$, $\sigma$ and $\sigma ^*$ were introduced above. Assuming identical return parameters for all organ-draining lymph nodes, $\mu _{\text {LN}}$, Eqs. (9) and (10) can be rewritten in the form used in the global sensitivity analysis eFAST (see “Global sensitivity analysis”):

11 $$    \begin{aligned} \begin{aligned} \frac{N^*_{\text {SLT}}}{N_{\text {SLT}}} = \kappa \frac{ V_{\text {LN}_{\text {TOT}}} + \frac{1}{\mu _{\text {LN}}} {\tilde{V}}_{\text {LN}_{\text {TOT}}} \left( 1 + (\sigma ^* - 1) \frac{{\tilde{V}}_{\text {liver}}}{\sum _{\begin{array}{l} i \notin \{ \text {spleen,PC,} \\ \text {RA} {\&} \text {RV,LA} {\&} \text {LV} \} \end{array}} {\tilde{V}}_i} \right) }{ V_{\text {spleen}} + \frac{1}{\mu _{\text {spleen}}}{\tilde{V}}_{\text {spleen}} + V_{\text {LN}_{\text {TOT}}} + \frac{1}{\mu _{\text {LN}}} {\tilde{V}}_{\text {LN}_{\text {TOT}}} \left( 1 + (\sigma - 1) \frac{{\tilde{V}}_{\text {liver}}}{\sum _{\begin{array}{l} i \notin \{ \text {spleen,PC,} \\ \text {RA} {\&} \text {RV,LA} {\&} \text {LV} \} \end{array}} {\tilde{V}}_i} \right) }, \end{aligned} \end{aligned}$$

12 $$\begin{aligned} \frac{{\bar{C}}^*_{\text {SLT}}}{{\bar{C}}_{\text {SLT}}} = \left. \frac{N^*_{\text {SLT}}}{N_{\text {SLT}}} \Bigg / \frac{{\tilde{V}}_{\text {LN}_{\text {TOT}}}}{ {\tilde{V}}_{\text {LN}_{\text {TOT}}} + {\tilde{V}}_{\text {spleen}} } \right. , \end{aligned}$$

where $V_{\text {LN}_{\text {TOT}}}$ and ${\tilde{V}}_{\text {LN}_{\text {TOT}}}$ is the total blood and organ volume of lymph nodes, respectively. The derivation again may be found in the Supplementary Material.

### In silico splenectomy—simulation

The effect of in silico splenectomy on T cell distribution across organs can be evaluated in two ways. Firstly, the ODE system can be solved explicitly for a set of chosen input parameters to compare the resulting pre- and post-splenectomy T cell distribution across all organs. To evaluate the post-splenectomy T cell distribution, in silico splenectomy can be simulated by setting the blood flow through the spleen, $B_{\text {spleen}}$, to zero, and adjusting the blood flow values for the remaining organs, according to the expected blood flow scaling (the derivation of blood flow changes post-splenectomy can be found in Supplementary Material). Alternatively, the steady-state model reduction (see “Steady-state model reduction”) can be utilised to evaluate the post- to pre-splenectomy cell concentration ratios, $\kappa$, $\kappa /\lambda$ and $\sigma ^*\kappa /\sigma$, which also accommodates blood flow changes, as well as considering the parameter groupings, $\lambda$, $\sigma$ and $\sigma ^*$. We have presented the analytically derived distributions in “Steady-state model reduction” section to highlight the advantage of model reduction given the prohibitively high dimensionality of the PBPK models.

The post- to pre-splenectomy cell concentration ratios, $\kappa$, $\kappa /\lambda$ and $\sigma ^*\kappa /\sigma$, and the parameter groupings, $\lambda$, $\sigma$ and $\sigma ^*$, were computed varying the input parameters $B_o$, $V_o$, ${\tilde{V}}_o$, $e_o$, $\mu _o$ for each organ, and $B_{\text {LN}_{\text {TOT}}}$, $V_{\text {LN}_{\text {TOT}}}$, ${\tilde{V}}_{\text {LN}_{\text {TOT}}}$, $e_{\text {LN}_o}$, $\mu _{\text {LN}_o}$ for lymph node compartments. Variability ranges for the anatomical parameters ($B_o$, $V_o$, ${\tilde{V}}_o$, $B_{\text {LN}_{\text {TOT}}}$, $V_{\text {LN}_{\text {TOT}}}$, ${\tilde{V}}_{\text {LN}_{\text {TOT}}}$) were defined as described in “Parameterisation” section, while extravasation and return parameters ($e_o$, $\mu _o$, $e_{\text {LN}_o}$, $\mu _{\text {LN}_o}$) were varied uniformly between 0 and 1. Extravasation, $e_{\text {LN}_o}$, and return parameters, $\mu _{\text {LN}_o}$, were assumed identical across all lymph node compartments for each parameter sampling iteration. Results with no restrictions on those parameters did not differ significantly, and are, therefore, not presented. This parameter space was sampled 1,000,000 times by randomly sampling each input parameter value from a uniform probability distribution, defined by those variability ranges. For each iteration, the post- to pre-splenectomy cell concentration ratios, $\kappa$, $\kappa /\lambda$ and $\sigma ^*\kappa /\sigma$, and the parameter groupings, $\lambda$, $\sigma$ and $\sigma ^*$, were calculated based on the randomly sampled parameter set. Means and 95% confidence intervals were computed for each resulting distribution.

To ensure convergence, all ratios and parameter groupings were evaluated for different numbers of sampling points in the parameter space, starting from 1000, and increasing the number of sample points by a factor of 10. All ratios and parameter grouping exhibited convergence in their means and confidence intervals for 1,000,000 sampling points. While this number is relatively small compared to the number of model parameters, the convergence stems from diminished sensitivity due to the structure of the considered functions [see Eq. (9) as an example], and the number of parameter samples is thus observed to be sufficient.

### Global sensitivity analysis

A global sensitivity analysis was performed on the post- to pre-splenectomy total T cell localisation ratio (Eq. 11) and the post- to pre-splenectomy net T cell concentration ratio (Eq. 12), to identify the most influential parameters. A variance-based method, extended Fourier amplitude sensitivity testing (eFAST), was implemented using the Matlab code developed and made available online by Kirschner Lab, University of Michigan^42^. The originally published code was first used to reproduce results of sensitivity analysis on the ODE model of HIV-CD4$^+$ T cells interaction by Marino et al.^43^ to ensure reliability.

Due to eFAST scaling constraints with respect to the number of parameters, simply sampling from all parameters in the model is far from feasible; instead we take advantage of the fact the ratios of interest in Eqs. (11) and (12) can be written in terms of the lumped parameters $\kappa$, $\sigma$ and $\sigma ^*$ together with a relatively small number of other parameters to be varied: the spleen return parameter $\mu _{\text {spleen}}$, the spleen organ and blood volumes, the total lymph node volume and lymph node blood volume and the liver organ volume. As previously, the lymph node return rates for each organ, $\mu _{\text {LN}_o}$, were assumed identical across all lymph node compartments, as was the sum of lymph node draining organ volumes.

**Figure 5:**
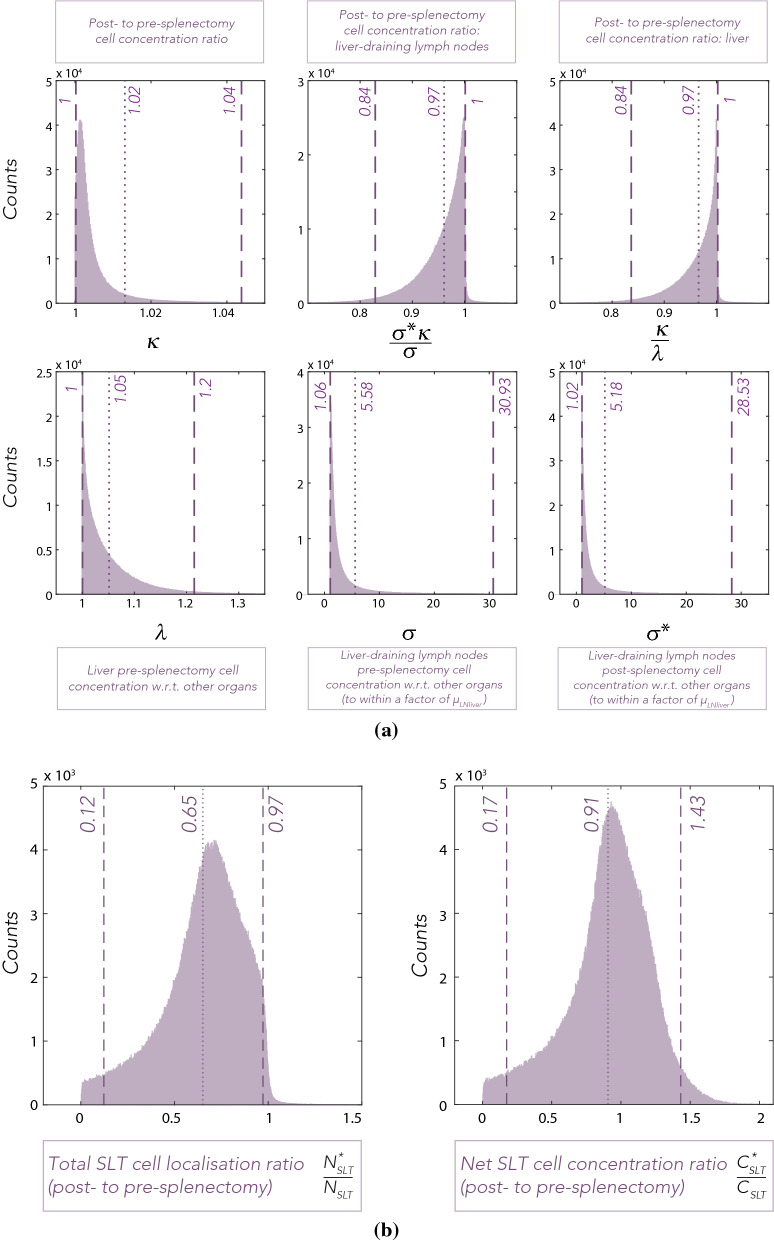
Distributions of (**a**) post- to pre-splenectomy cell concentration ratios: $\kappa$, $\sigma ^*\kappa /\sigma$ and $\kappa /\lambda$, as well as $\lambda$, $\sigma$ and $\sigma ^*$, and (**b**) the post- to pre-splenectomy total T cell localisation ratio and the net T cell concentration ratio in the SLT. Dotted and dashed lines correspond to means and 95% confidence intervals of the plotted distributions. See “Parameterisation” and “In silico splenectomy—simulation” sections for details on parameters.

The sensitivity analysis is thus considered with a sampling of the lumped parameters from their distributions in Fig. 5a, cut off at the 95% confidence interval, together with the other parameters to be varied sampled uniformly, as detailed in “In silico splenectomy—simulation” section and the supplementary information. However the eFAST algorithm is designed for uniform sampling, in contrast to the distribution of the lumped parameters $\kappa , ~\sigma$ and $\sigma ^*$. Thus for example, we thus uniformly sampled $\kappa _U :=f(\kappa )$, within eFAST rather than $\kappa$, where the continuous bijection $f(\cdot )$ is defined uniquely by the constraint that when $\kappa _U$ is uniform then $\kappa$ is distributed as in Fig. 5a. Then by applying the function inverse $f^{-1}$ to the the precursor, $\kappa _U$, we have $\kappa$ is generated according to its distribution in Fig. 5a, with analogous procedures for the lumped parameters $\sigma$, $\sigma ^*$. In particular parameter value sets for the uniform precursors of the lumped parameters $\kappa$, $\sigma$, $\sigma ^*$ and the other parameters of interest in Eqs. (11) and (12) were generated using Fourier frequency curves through the variability ranges. Each of these parameters was sampled $N_S =$ 1920 times with a frequency of $\omega _i =$ 49 from a uniform probability distribution^44^. Due to the symmetry properties of trigonometric functions, the sampling sinusoidal function eventually repeats the same samples. To avoid this, the sampling algorithm was repeated $N_R =$ 5 times^44^. The resampling produced 5 different search curves, specified by introducing a random phase shift into each sinusoidal function.

By assigning a unique frequency to each input parameter, the eFAST algorithm calculates the unique contribution of each parameter in determining the model output. The analysis yields two indices for each parameter: (i) a first-order sensitivity index, $S_i$, representing the proportion of an output’s variance that is due only to a given parameter (first-order contributions), and (ii) total-order sensitivity index, $S_{Ti}$, calculated from the proportion of an output’s variance due to all parameters except the one in question (higher-order contributions). The difference between the total-order and the first-order sensitivity indices gives a measure of the impact of the interactions between the parameters.

The eFAST method artificially produces small but non-zero sensitivity indices for parameters which the model is completely independent of, due to aliasing and interference effects, as well as assumptions used to calculate total-order sensitivity indices^43^. A dummy parameter with an arbitrary value range was considered to provide a baseline for statistical comparison, as proposed by Marino et al.^43^. Thus, a total-order sensitivity index less than or equal to that of the dummy parameter is, therefore, not considered substantially different from zero.

The resampling of search curves, which is originally used for more efficient parameter sampling, produces different combinations of parameter values for each curve, and, hence, slightly different sensitivity measures $S_i$ and $S_{Ti}$. The repeated measures were used to perform a two-sample t-test to compare the sensitivity indices of each parameter with the corresponding indices assigned to the dummy parameter^43^. The t-test method assigns a p-value to each sensitivity index, which provides an additional way to determine whether the index is significantly different from the index calculated from the dummy parameter, and, thus, whether there is sensitivity to the parameter.

Finally, we note a caveat with this procedure: the lumped parameters $\sigma$ and $\sigma ^*$ depend on $\mu _{\text {spleen}}$, but $\sigma$, $\sigma ^*$ and $\mu _{\text {spleen}}$ are varied independently during the sensitivity analysis. However, $\sigma$ and $\sigma ^*$ depend on $\mu _{\text {spleen}}$ very weakly with an error on the scale of the reciprocal of the number of organs as $\mu _{\text {spleen}}$ only occurs in additive combination of a summation over the organs. Hence the impact of not accommodating this correlation in the sampling is also weak. An analogous caveat applies for $\kappa$ and its dependence on parameters that are also varied in the sensitivity analysis. That this is a weak effect is not immediately observed *a priori*, as with $\sigma$ and $\sigma ^*$, but it can be confirmed *a posteriori* as we briefly consider in the presentation of results.

## Results

The immune cell trafficking model was used to examine predicted distributions of T cells across different SLT compartments—lymph nodes and the spleen. Since the spleen comprises a major part of the SLT, we sought to investigate how splenectomy affects the T cell distribution in other organs, particularly, the lymph nodes.

### Post- and pre-splenectomy comparisons

The response of the system to splenectomy can be qualitatively and quantitatively analysed by considering a reduced framework. Despite the high complexity of the trafficking model (101 parameters), the steady-state model reduction demonstrated that post- to pre-splenectomy cell concentration ratios across all organ compartments can be described by four variables. It further showed that, upon the removal of the spleen, all but two organ compartments result in the same relative change in the steady-state T cell concentration. The exceptions are the liver and liver-draining lymph node compartments, where a lower relative increase in cell concentration is expected, due to the fact that all T cells exiting the spleen flow directly to the liver.

The expected post- to pre-splenectomy cell concentration ratios were expressed as functions of the model input parameters and their distributions were evaluated based on 1,000,000 uniform samplings in the parameter space. All parameters were randomly sampled from uniform probability distributions, defined by the variability ranges (see section entitled “In silico splenectomy—simulation” and Supplementary Material). The ratios’ and variables’ distributions were plotted as histograms in Fig. 5a with evaluated means and 95% confidence intervals indicated.

The obtained post- to pre-splenectomy T cell concentration ratios were characterised by relatively narrow distributions. This was quantitatively illustrated by the 95% confidence intervals (Fig. 5a) and demonstrates that the simulated relative change in T cell exposure post-splenectomy in different tissues can be estimated despite the unknown tissue-specific entry and exit rates, as well as the large interpersonal and temporal variability in the physiological parameters. For instance, the volume of muscle mass can vary significantly between individuals (muscle mass can reach 65.1% of body mass in athletes and bodybuilders^35^), and blood flow to muscles and blood volume in the tissue increases significantly during exercise (the total blood flow can reach 30–35 L/min in elite endurance athletes^38^, roughly 6 times higher than the baseline^32^). Yet the relative change in T cell exposure post-splenectomy in the majority of organ and organ-draining lymph node compartments, $\kappa$, resulted in the mean of 1.02 and 95% confidence interval of 1–1.04. The predicted T cell exposure ratio was more broadly distributed for the two remaining compartments, the liver ($\kappa /\lambda$) and liver-draining lymph node compartments ($\sigma ^*\kappa /\sigma$), both with the means of 0.97 and 95% confidence intervals of 0.84–1.

The negligibly low mean relative increase of T cell exposure, $\kappa$, suggests little to no change in the lymph node cellular capacity post-splenectomy to account for the loss of the spleen. The post- to pre-splenectomy total cell localisation in SLT was evaluated as a ratio of the total number of T cells localising to all lymph node compartments post-splenectomy to the total number of cells localising to both the spleen and all lymph node compartments pre-splenectomy (Eq. 9). Since a quantity of concentration is often used in the clinic, a similar ratio of net T cell concentration in SLT was evaluated (Eq. 10). However, as concentrations are not additive, the net T cell concentration in SLTs pre-splenectomy (post-splenectomy) has been defined as the number of T cells localising to all lymph node compartments and the spleen (all lymph node compartments) per unit volume of secondary lymphoid tissue: lymph nodes and the spleen (lymph nodes). As a result, a ratio defined in this manner can be interpreted as a relative change in the number of T cells per unit mass of SLT.

### Cell localisation and concentration ratios before and after splenectomy

The localisation and concentration ratio distributions were plotted as histograms (Fig. 5b), with the means and 95% confidence intervals indicated on the graphs. In silico splenectomy resulted in an average post- to pre-splenectomy total T cell localisation ratio of 0.65 (95%CI 0.12–0.97), indicating a statistically significant 35% reduction in T cells localising to SLT post-splenectomy. The distribution has been characterised by a wide 95% confidence interval compared to the standard T cell concentration ratio, such as $\kappa$ (see Fig. 5a).

The corresponding distribution of the net SLT T cell concentration ratio was characterised by a mean of 0.91, with a confidence interval of 0.17–1.43. Contrasting the results of localisation ratio with the concentration ratio shows that, although, on average, the net concentration in SLT is reduced, the resulting relative concentration decrease is not statistically significant. Consequently, the analysis showed a considerable difference in the T cell localisation but no substantial difference in the net concentration post-splenectomy.

#### Sensitivity of cell localisation and concentration ratios pre- and post- splenectomy

**Figure 6:**
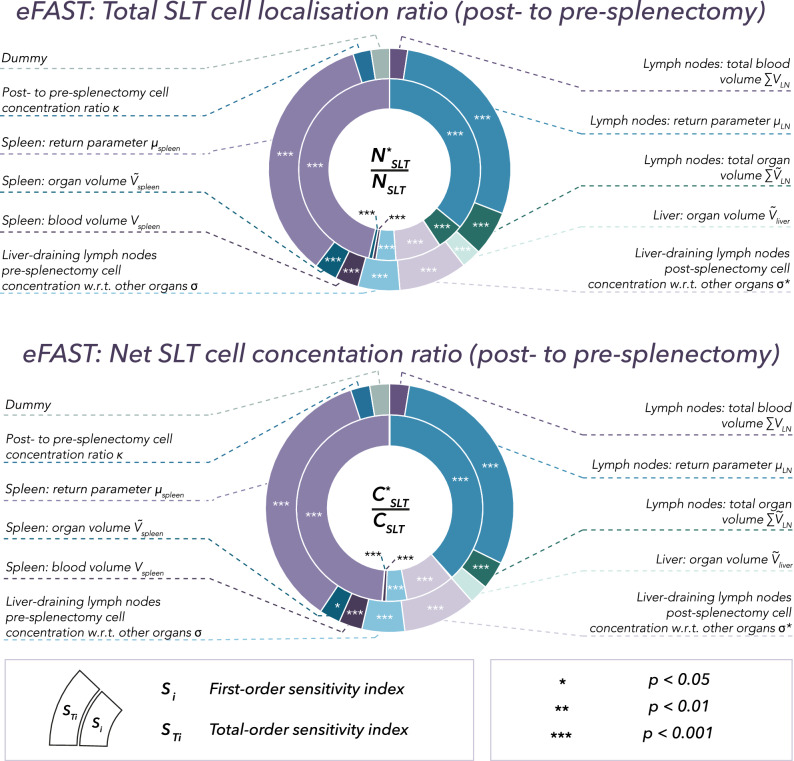
Results of the global sensitivity analysis eFAST on the post- to pre-splenectomy total T cell localisation to SLT ratio (upper) and the post- to pre-splenectomy net T cell concentration in SLT ratio (lower). The inner (outer) ring indicates the proportional size of individual sensitivity indices (total sensitivity indices). A dummy parameter is used as a baseline for statistical comparison and a p-value was assigned to each sensitivity index using a two-sample t-test^43^. Parameters with statistically significant sensitivity indices were labelled according to the assigned p-value ($$^*$$,$$^{**}$$,$$^{***}$$). The eFAST sensitivity analysis considered with a sampling of the lumped parameters $$\kappa $$, $$\sigma $$, $$\sigma ^*$$ from their distributions in Fig. 5a, cut off at the 95% confidence interval, together with the other parameters to be sampled uniformly, as detailed in “Global sensitivity analysis” section, with further details on parameter distributions in “Parameterisation” and “In silico splenectomy—simulation” sections.

A variance-based global sensitivity analysis, eFAST, was performed to identify the parameters driving the wide distributions of the post- to pre-splenectomy total T cell localisation ratio (Eq. 9) and the post- to pre-splenectomy net T cell concentration ratio (Eq. 10) (Fig. 6). Both the post- to pre-splenectomy total T cell localisation ratio and the post- to pre-splenectomy net T cell concentration ratio were primarily dependent on the return parameters of the spleen and lymph nodes, $\mu _{\text {spleen}}$ and $\mu _{\text {LN}}$ (varied separately). The strong sensitivity of the localisation and concentration ratios to the return parameters, $\mu _{\text {spleen}}$ and $\mu _{\text {LN}}$, stems from the definition of these parameters, which determine the rate at which T cells leave the associated interstitial compartments (see Eq. 2). As such, the return parameters, $\mu _{\text {spleen}}$ and $\mu _{\text {LN}}$, are the primary parameters affecting the accumulation of T cells in steady state in SLT compartments and, thus, the resulting relative T cell concentration SLT following the redistribution of T cells from the spleen. The eFAST analysis also indicated slight sensitivity to the variables $\sigma$ and $\sigma ^*$, which, to within a factor of $\mu _{\text {LN}_{\text {liver}}}$, define the steady-state T cell concentration in liver-draining lymph nodes relative to the steady-state vasculature concentration (equal for all organs except the liver) pre- and post-splenectomy, respectively. Although small, the statistically significant sensitivity indices associated with the variables $\sigma$ and $\sigma ^*$ highlight the relevance of the specific anatomical structure of hepatic circulation in lymphocyte trafficking modelling. As a whole, both the localisation and net concentration ratio yielded consistent and comparable sensitivity indices, with the net concentration ratio being moderately less sensitive to the spleen and total lymph nodes volumes, as expected from the ratio of Eqs. (9) and (10).

Finally, as mentioned in “Global sensitivity analysis” section the dependence of the lumped parameter $\kappa$ on the other sampled parameters has been neglected. However, this neglect serves to increase the variation of $\kappa$ (e.g., fixing a parameter within $\kappa$ reduces, or at least does not increase, the range it can acquire compared to the distribution in Fig. 5a, where the same parameter was free to vary in its sampling). However even with an artifact of increased variation the sensitivity scores of $\kappa$ in Fig. 6 are small compared to the scores of the more sensitive parameters and do not exceed those associated with the dummy parameter. Thus the results show that the impact of the lumped parameter $\kappa$ is below the level of meaningful classification in this sensitivity analysis regardless of the above-mentioned caveat in its sampling.

## Discussion

Recent cohort study data reporting a marked increase in the haematological malignancies risk in splenectomised subjects^13,14^, suggests that splenectomy leads to a loss of effective immunosurveillance of blood-borne malignant cells, as the remaining secondary lymphoid tissue has been predicted to not have the sufficient capacity to compensate. Given the natural trafficking pattern of T cells to the spleen and lymph nodes, the primary aim of this study has been to examine how splenectomy affects T cell distribution across secondary lymphoid tissue in human.

Using a PBPK modelling approach, a previously developed immune cell trafficking framework^27^ was adopted, refined and specialised to predict T cell biodistribution in the presence and absence of the spleen. The effect of in silico splenectomy on lymph node tissue exposure was investigated to estimate to what extent the remaining secondary lymphoid tissue has the potential capacity to compensate for the loss of immune functionality, normally mediated by the spleen. By utilising the model’s structure, post- to pre-splenectomy cell concentration ratios were derived analytically and simulated across the parameter space. The variability in the input parameters was carefully considered to account for possible values of entry and exit rates of T cells across all tissues, as well as interpersonal and temporal differences in anatomical and physiological characteristics.

The resulting simulations of the relative changes in the organ T cell concentration were characterised by narrow distributions, despite large variability in the input parameters. All tissues and tissue-specific groups of lymph nodes, except the liver and the liver-draining lymph nodes, exhibited an equal post- to pre-splenectomy cell exposure, $\kappa$, with a mean of 1.02 (95%CI 1–1.04). Since the mean relative increase in lymph node cell exposure was negligibly low, the post- to pre-splenectomy total T cell localisation and net T cell concentration in secondary lymphoid tissue were also evaluated. Given the uniform sampling across parameter space, the net concentration of T cells in secondary lymphoid tissue had a mean of 91% in asplenic patients compared to normal patients (0.91, 95%CI 0.17–1.43) and the net localisation of T cells in secondary lymphoid tissue was reduced by 35% (0.65, 95%CI 0.12–0.97). This indicates that, while in silico splenectomy produces a minimal change in the secondary lymphoid tissue exposure (concentration), it significantly reduces the total number of T cells localising to secondary lymphoid tissue and, thus, possible activation events. In turn, this suggests that remaining lymph nodes do not have sufficient trafficking thoroughfare to compensate for lost T cell traffic within the spleen, even before detailed functional differences in the spleen and lymph nodes are considered. These results appear to corroborate the clinical data that asplenic patients may be more susceptible to haematological malignancies or do worse with the conditions than patients with full spleen function^13,14^. The results further highlight that compensatory pathways to mitigate the loss of the spleen are unlikely.

As with all models, the immune cell trafficking model was constructed based on certain assumptions, which impose limitations on the interpretation of results. Firstly, the model treats lymph node tissue as 18 separate organ-specific groups of lymph nodes, and each group is represented as a ‘lumped’ compartment. In reality, lymph nodes are widely present throughout the human body and often arranged in chains, which can drain more than one organ (particularly in mesentery). Secondly, lymph nodes were partitioned into groups across the body based on the volume of the organ they drain from. The data on lymph node whole-body distribution and lymphatic drainage pathways is limited, largely relying on 19th-century cadaver studies^45^, which have been reported to contrast with some recent clinical data^9^. Further data on the lymphatic system anatomy is needed, but the current state of knowledge suggests that both the size and number of lymph nodes increases with the body mass between different species^46^. The assumption that the number of lymph nodes, and consequently the total volume of lymph nodes, will scale with organ volume has therefore been chosen as a parsimonious working approximation. To the best of our knowledge, no previously published PBPK model of immune cell trafficking contained more than three lymph node compartments^24,25^. Integrating organ-specificity into a framework provides a more physiologically accurate description, albeit still being a significant simplification of the anatomy of the lymphatic system. However, whether such complexity is necessary to accurately depict T cell dynamics is beyond the scope of this paper.

Although the impact of splenectomy on T cell distribution could, in theory, be modelled with a smaller, though reasonable, trafficking framework, the use of a large mathematical model, as presented here, does in fact offer several advantages, given it dramatically simplifies. The complexity of the model affords the possibility of examining the research question without *a priori* simplifying components of the model, which might be necessary for understanding the mechanism of splenectomy but would not otherwise be captured using a simpler framework, for instance, the anatomy of the hepatic circulation. The steady-state model reduction approach additionally provides structural and biological insight that is not feasible from numerical simulations only. Noting the reduction in the size of the parameter space on model simplification, with the concomitant removal of the curse of dimensionality, the computational advantage is substantial. For example, it renders a global sensitivity analysis feasible and allows a verification that sufficient sampling of the parameter space has been performed, in turn enabling a comprehensive survey of the prospective features that may, or may not, have not an impact on the T cell redistribution post-splenectomy, while fully accommodating physiological features such as the hepatic circulation.

The impact of splenectomy on T cell trafficking has so far been addressed experimentally across different species (mice^22^, rats^47^, sheep^48^, pigs^49^) and across different time periods of observation. Experimental and theoretical distribution profiles of primed T cells from five tissues in tumour-bearing non-splenectomised and splenectomised mice suggested a compartmental shift in lymphocyte distribution following splenectomy^22^. Similarly to our model’s predictions, the retention of transferred cells in the liver was greater in non-splenectomised mice compared to splenectomised animals^22^.

Despite the relatively high retention of cells within the spleen, splenectomy had a negligible effect on tumour lymphocyte recruitment^22^. Another short timescale experiment, with splenectomised pigs, showed that labelled lymphocytes exit the peripheral blood at much slower rate than in non-splenectomised animals^49^. The data collected from isolated perfused pig spleen demonstrated that lymphocytes recirculate through the spleen at a rapid rate of up to $270 \times 10^9$ lymphocytes per day in living pigs^49^, highlighting the role of the spleen for recirculating T cells.

However, a shorter time period between splenectomy and data collection may highlight many transient effects, including trauma, which we are not accommodating, and we similarly do not directly consider the presence of a tumour in this study. Hence, we only consider how the overall framework and quantitative predictions compare with relatively longer experiments. One instance is a study of rats, with a follow-up of 15 months after the removal of the spleen. While there was a differential effect on different subsets of T cells, the overall numbers of T cells were comparable in splenectomised and control group of animals^47^. Similarly, a study on 2-year-old neaonatally-splenectomised sheep did not identify significant changes in the blood profile^48^. These results appear in agreement with the qualitative features of the model’s predictions across parameter space in that the relative predicted increase of T cells within the vasculature compartments is very modest and may not be resolvable in practice. While consistent, these qualitative comparisons of model prediction and observation need to be interpreted with suitable caution, particularly in the light of difficulties with interspecies translation and the question of whether the number of T cells in the blood reflects the whole pool of recirculating T cells. Furthermore, while, to the best of our knowledge, no clinical or preclinical data has been published suggesting a potential long-term compensatory mechanism affecting T cell numbers or trafficking dynamics post-splenectomy, such a mechanism cannot be ruled out.

The spleen plays an important role in the generation of adaptive immune responses, but the impact of splenectomy on immunocompetence has not been fully understood yet. The predicted lack of significant relative difference in the T cell localisation upon virtual removal of the spleen predicts an insufficient cellular and trafficking capacity of secondary lymphoid tissue to compensate for immune functions normally mediated by the spleen. The implied deficit is particularly noteworthy given the clinical data suggesting that asplenic patients are at higher risk of incidence and mortality of haematological malignancies^13,14^. Although the direct link between splenectomy and cancer is yet to be established, detailed statistical analyses of the clinical data revealed that the risk of malignancies remained significantly elevated in analyses limited to trauma patients as well as those excluding patients with an autoimmune disease prior to splenectomy^13^.

The mechanisms for splenectomy increasing the risk of many haematological malignancies over solid tumours^13,14^ are not scientifically demonstrated. The physiological characteristics of the spleen may make the organ a more suitable site for the detection of malignant cells persisting in the bloodstream, as would be the case for haematological malignancies. The relatively higher perfusion of the spleen (0.87 min$^{-1}$^27^) compared to lymph nodes (0.41 min$^{-1}$)^27^) also contributes to the noticeably higher estimated maximal cell delivery rate to the spleen (27.9 cells min$^{-1}$ mm$^{3}$^27^) in comparison to lymph nodes (13  cells min$^{-1}$ mm$^{3}$^27^), and thus enhanced immunosurveillance with a spleen. The physiological and anatomical differences between the SLT components underlie the predicted reduction in the mean number of T cells localising in the SLT in the absence of the spleen. Differences in spleen versus lymph node lymphocyte activation may additionally play a more crucial role in the context of specific disease pathophysiologies or processes, such as the recognition and clearance of blood-borne leukaemic or metastatic cells from the blood compartment, as opposed to abnormal cells comprising solid tumours.

While CD8$^+$ T cells comprise a major group of anti-tumour effector cells, other types of immune cells also play the role in the elimination of cancer. Circulating NK cells, which possess cytotoxic abilities similar to CD8$^+$ T cells but lack CD3 and T cell receptors (TCRs), are largely found in blood, bone marrow and lymphoid tissues, such as the spleen^50^. Although their relevance in the immune surveillance of human solid tumours is still not fully elucidated, a wide range of pre-clinical and clinical data suggests that NK cells are particularly important for the immune response to haematological malignancies^51^. Given the number and activity of NK cells have been correlated with prognosis in several haematological malignancies and that the spleen serves as a large reservoir of NK cells^51^, it is plausible that the impact of splenectomy on the NK cell trafficking may further contribute to the observed increased risk of many haematological malignancies over solid tumours in splenectomised patients^13,14^.

Another example of a T cell subset with a recently demonstrated role in oncology are resident memory, $\hbox {T}_{{RM}}$, cells, identified a decade ago as non-recirculating tissue-resident T cells^52^. They are now considered to represent a separate T cell lineage due to the expression of specific markers of residency and the lack of molecules that enable egress from the tissue and subsequent migration to lymph nodes^52^. $\hbox {T}_{{RM}}$ cells are also characterised by a distinct differentiation profile, dependent on antigen presentation, certain cytokines and local inflammation^53^. Similarly to central memory T ($\hbox {T}_{{CM}}$) cells, $\hbox {T}_{{RM}}$ cells are long-lived. However, in contrast to $\hbox {T}_{{CM}}$ cells, $\hbox {T}_{{RM}}$ cells are restricted to specific tissues, such as the skin and lung, providing superior immunity compared to circulating memory cells^53^. Interestingly, although $\hbox {T}_{{RM}}$ cells can be generated from naïve T cells primed in the LN, other antigen-experienced cells, such as central memory T cells ($\hbox {T}_{{CM}}$) and effector CD8$^+$ T cells, can also differentiate into $\hbox {T}_{{RM}}$ cells, suggesting certain plasticity of the pool of memory T cells^53^. While the T cell trafficking model presented here represents an exchange of recirculating T cells, and, thus, cannot serve as a tool to draw conclusions regarding tissue-resident T cells, the role of non-recirculating T cells in the immunosurveillance against malignant cells in peripheral tissues may further contribute to the lower solid tumour incidence compared to that of haematological malignancies in asplenic population but remains to be established. In the absence of further clinical data, however, PBPK models can serve as a guide to determining the likely effect of changing the physiological status of the body on the trafficking of not only T cells, but also other types of cells, such as B, NK cells, and cellular therapies. Such cell-kinetic modelling frameworks, in turn, allow the simulation of clinical scenarios of interest and quantifying the relative exposure of therapy to the spleen, lymph nodes or a tumour, which may lead to further insight into the improved design of immunotherapies, while highlighting the potential impact of confounding factors, such as the case of splenectomy studied here.

## Supplementary Information

Supplementary Information.
