# Supplementary material for: Predicted limited redistribution of T cells to secondary lymphoid tissue correlates with increased risk of haematological malignancies in asplenic patients
Source: Sci Rep. 2021 Aug 12;11:16394. doi: 10.1038/s41598-021-95225-x (PMC8360980; doi:10.1038/s41598-021-95225-x)
Supplement: Supplementary file 1 — Supplementary Information. [file 41598_2021_95225_MOESM1_ESM.pdf]

Predicted limited redistribution of T cells to secondary lymphoid tissue correlates with increased risk of haematological malignancies in asplenic patients

Aleksandra E. Kmieciak[1,*], Liam V. Brown[1], Mark C. Coles[2], Jonathan Wagg[3], Alex Phipps[4,], and Eamonn A. Gaffney[1]**

[1]Wolfson Centre for Mathematical Biology, Mathematical Institute, University of Oxford, Andrew Wiles Building, Woodstock Rd, Oxford OX2 6GG, United Kingdom
[2]Kennedy Institute of Rheumatology, University of Oxford, Roosevelt Dr, Headington, Oxford OX3 7FY, United Kingdom
[3]AC Immune, EPFL Innovation Park, 1015 Lausanne, Switzerland
[4]Pharmaceutical Sciences - Clinical Pharmacology, Roche Innovation Centre Welwyn, 6 Falcon Way, Shire Park, Welwyn Garden City AL7 1TW, United Kingdom
[*]kmieciak@maths.ox.ac.uk
[**]Presently at Clinical Pharmacology and Quantitative Pharmacology - Oncoloy, AstraZeneca, Cambridge CB2 0AA, United Kingdom

1 Full ODE system

The ODE system, representing the exchange of any cell population (here applied to T cells) between the heart and organs, has been introduced in Section entitled" Model Overview", in the Main Text. The model, based on Figure 2 (right) in the Main Text, describes the number of T cells in each organ compartment as a function of time. Recall the rate of change in the number of T cells in the vasculature of an organ, o, is given by:

$$\frac{dN_o}{dt} = V_o \frac{dC_o}{dt} = B_o \left(C_{\mathrm{LA\&LV}} - C_o\right), \tag{S.1}$$

where V_o is the blood volume of the organ, o, B_o is the blood flow of the organ, o, while $C_{\mathrm{LA\&LV}}$ is the concentration of T cells within the left atrium, left ventricle, aorta and large arteries between organs and the heart. The rate of change in the number of T cells in the interstitium of an organ, o, is given by:

$$\frac{d\tilde{N}_o}{dt} = \tilde{V}_o \frac{d\tilde{C}_o}{dt} = e_o B_o \left(C_o - \mu_o \tilde{C}_o\right), \tag{S.2}$$

where $\tilde{V}_o$ is the volume of the organ, o, $\tilde{C}_o$ is the interstitial concentration of T cells, e_o and μ_o are the extravasation and return parameters, respectively. The first term describes a proportion of T cells, $e_o B_o$ extravasating into the interstitial space of organ, o, and the second term represents a proportion of T cells, $e_o \mu_o B_o$, flowing via lymphatics into organ, o, -draining lymph nodes (see Figure 3 in the Main Text).

Each group of organ-draining lymph nodes is represented by a vasculature sub-compartment (the blood supply to the lymph node) and the interstitial sub-compartment, where T cells from the interstitium of organ, o, are drained via lymphatics (see Figure 3 in the Main Text). The rate of change in the number of T cells in the lymph nodes' blood supply compartment, analogously to Equation (S.1), is given by:

$$\frac{dN_{\mathrm{LN}_o}}{dt} = V_{\mathrm{LN}_o} \frac{dC_{\mathrm{LN}_o}}{dt} = B_{\mathrm{LN}_o} \left(C_{\mathrm{LA\&LV}} - C_{\mathrm{LN}_o}\right), \tag{S.3}$$

where V_{LN_o} is the blood volume of lymph nodes draining from an organ, o, B_{LN_o} is the blood flow through these lymph nodes, and $C_{\mathrm{LA\&LV}}$ is the concentration of T cells within the left atrium, left ventricle, aorta and large arteries between organs and the heart. The rate of change of T cells in the interstitial lymph node group compartment accounts for the contribution from the lymph nodes' vasculature and, additionally, the flow of interstitium drained cells through organ, o:

$$\frac{d\tilde{N}_{\mathrm{LN}_o}}{dt} = \tilde{V}_{\mathrm{LN}_o} \frac{d\tilde{C}_{\mathrm{LN}_o}}{dt} = e_{\mathrm{LN}_o} B_{\mathrm{LN}_o} \left(C_{\mathrm{LN}_o} - \mu_{\mathrm{LN}_o} \tilde{C}_{\mathrm{LN}_o}\right) + e_o \mu_o B_o \left(\tilde{C}_o - \mu_{\mathrm{LN}_o} \tilde{C}_{\mathrm{LN}_o}\right), \tag{S.4}$$

where $\tilde{V}_{\mathrm{LN}_o}$ is the volume of draining lymph nodes and μ_{LN_o} is the fraction of cells returning to the systemic circulation per unit blood flow. The first term describes the flow of T cells from the lymph node blood supply sub-compartment into the interstitium and the proportion of the T cells that have extravasated leaving the interstitium. The second term represents T cells drained from an organ, o, interstitium and the proportion of the drained T cells being returned to the systemic circulation. Note that mediastinal lymph nodes, which drain both the vasculature of the heart and lungs, are an exception. The rate of change in the number of T cells in the blood supply compartment, analogously to Equation (S.3), is given by:

$$\frac{dN_{\mathrm{LN}_{\mathrm{med}}}}{dt} = V_{\mathrm{LN}_{\mathrm{med}}} \frac{dC_{\mathrm{LN}_{\mathrm{med}}}}{dt} = B_{\mathrm{LN}_{\mathrm{med}}} \left(C_{\mathrm{LA\&LV}} - C_{\mathrm{LN}_{\mathrm{med}}} \right), \tag{S.5}$$

where $V_{\mathrm{LN}_{\mathrm{med}}}$ is the blood volume of lymph nodes draining from the vasculature of the heart and lungs, $B_{\mathrm{LN}_{\mathrm{med}}}$ is the blood flow through these lymph nodes, and $C_{\mathrm{LA\&LV}}$ is the concentration of T cells within the left atrium, left ventricle, aorta and large arteries between organs and the heart. The rate of change of T cells in the interstitial lymph node compartment accounts for the dual drainage of T cells (from the heart and lungs blood supply) in addition to T cells extravasating from the vasculature lymph node compartment:

$$\begin{aligned} \frac{d\tilde{N}_{\mathrm{LN}_{\mathrm{med}}}}{dt} = \tilde{V}_{\mathrm{LN}_{\mathrm{med}}} \frac{d\tilde{C}_{\mathrm{LN}_{\mathrm{med}}}}{dt} &= e_{\mathrm{LN}_{\mathrm{med}}} B_{\mathrm{LN}_{\mathrm{med}}} \left(C_{\mathrm{LN}_{\mathrm{med}}} - \mu_{\mathrm{LN}_{\mathrm{med}}} \tilde{C}_{\mathrm{LN}_{\mathrm{med}}} \right) \\ &+ \sum_{\substack{o \in \{\text{lungs b.s.,} \\ \text{heart b.s.}\}}} e_o \mu_o B_o \left(\tilde{C}_o - \mu_{\mathrm{LN}_{\mathrm{med}}} \tilde{C}_{\mathrm{LN}_{\mathrm{med}}} \right), \end{aligned} \tag{S.6}$$

where $\tilde{V}_{\mathrm{LN}_{\mathrm{med}}}$ is the volume of draining lymph nodes, $\mu_{\mathrm{LN}_{\mathrm{med}}}$ is the fraction of cells returning to the systemic circulation per unit blood flow, and *b.s.* stands for blood supply. The first term describes the flow of T cells from the lymph node blood supply sub-compartment into the interstitium and the proportion of the T cells that have extravasated leaving the interstitium. The second term represents T cells drained from the heart and lungs blood supply interstitium and the proportion of the drained T cells being returned to the systemic circulation.

The liver and liver-draining lymph nodes are further exceptions to Equations (S.1) – (S.4), which need to be modified to capture the specific anatomy of hepatic blood and lymph circulation. The framework treats the hepatic circulation separately from the blood received from the mesentery via the hepatic portal vein, *i.e.,* the blood flow through the liver as the hepatic circulation does not include the contribution from the portal vein (Table 2). Instead, the liver receives additional blood exiting the mesenteric organs: the stomach, spleen, pancreas, small and large intestines. Note that since the spleen lacks draining lymphatic vessels, the liver's vasculature receives both the T cells that have extravasated into the spleen and those that have not, and hence:

$$\begin{aligned} \frac{dN_{\mathrm{liver}}}{dt} = V_{\mathrm{liver}} \frac{dC_{\mathrm{liver}}}{dt} &= B_{\mathrm{liver}} \left(C_{\mathrm{LA\&LV}} - C_{\mathrm{liver}} \right) + e_{\mathrm{spleen}} \mu_{\mathrm{spleen}} B_{\mathrm{spleen}} \left(\tilde{C}_{\mathrm{spleen}} - C_{\mathrm{liver}} \right) \\ &+ \sum_{o \in \{\mathrm{mesentery}\}} B_o \left(1 - e_o \right) \left(C_o - C_{\mathrm{liver}} \right), \end{aligned} \tag{S.7}$$

where each term respectively describes: (i) T cells from the blood supply to the liver and returning to the systemic circulation, (ii) the flow and exit of T cells that have extravasated into the spleen and (iii) the flow and exit of T cells that have not extravasated from the mesenteric organs (the stomach, spleen, pancreas, small and large intestine) and have returned through the portal vein. The number of T cells in the liver's interstitium, therefore, changes in time according to:

$$\begin{aligned} \frac{d\tilde{N}_{\mathrm{liver}}}{dt} = \tilde{V}_{\mathrm{liver}} \frac{d\tilde{C}_{\mathrm{liver}}}{dt} &= e_{\mathrm{liver}} B_{\mathrm{liver}} \left(C_{\mathrm{liver}} - \mu_{\mathrm{liver}} \tilde{C}_{\mathrm{liver}} \right) \\ &+ \sum_{o \in \{\mathrm{mesentery}\}} B_o \left(1 - e_o \right) e_{\mathrm{liver}} \left(C_{\mathrm{liver}} - \mu_{\mathrm{liver}} \tilde{C}_{\mathrm{liver}} \right) \\ &+ e_{\mathrm{liver}} \left(C_{\mathrm{liver}} - \mu_{\mathrm{liver}} \tilde{C}_{\mathrm{liver}} \right) e_{\mathrm{spleen}} \mu_{\mathrm{spleen}} B_{\mathrm{spleen}}, \end{aligned} \tag{S.8}$$

where each term respectively represents: (i) the flow and drainage of T cells that have extravasated from the liver's blood supply, (ii) the flow and drainage of T cells that have not extravasated in the mesentery, returned to the liver's vasculature through the portal vein and subsequently extravasated into the liver interstitium (where mesentery refers to: the stomach, spleen, pancreas, small and large intestines) and (iii) the flow and drainage of T cells have extravasated in the spleen, returned to the liver's vasculature and subsequently extravasated into the liver interstitium.

The analogous equation for the liver-draining lymph nodes, LN_{liver}, is modified accordingly:

$$\begin{aligned}
\frac{d\tilde{N}_{\text{LN}_{\text{liver}}}}{dt} = \tilde{V}_{\text{LN}_{\text{liver}}}\frac{d\tilde{C}_{\text{LN}_{\text{liver}}}}{dt} &= e_{\text{LN}_{\text{liver}}}B_{\text{LN}_{\text{liver}}}\left(C_{\text{LN}_{\text{liver}}} - \mu_{\text{LN}_{\text{liver}}}\tilde{C}_{\text{LN}_{\text{liver}}}\right) \\
&+ e_{\text{liver}}\mu_{\text{liver}}B_{\text{liver}}\left(\tilde{C}_{\text{liver}} - \mu_{\text{LN}_{\text{liver}}}\tilde{C}_{\text{LN}_{\text{liver}}}\right) \\
&+ e_{\text{liver}}\mu_{\text{liver}}\left(e_{\text{spleen}}\mu_{\text{spleen}}B_{\text{spleen}} + \sum_{o\in\{\text{mesentery}\}} B_o\left(1-e_o\right)\right)\left(\tilde{C}_{\text{liver}} - \mu_{\text{LN}_{\text{liver}}}\tilde{C}_{\text{LN}_{\text{liver}}}\right),
\end{aligned} \tag{S.9}$$

where the second term describes the fraction of T cells from the liver's interstitium and the third term the flow of T cells that did not extravasate in the mesentery and entered the liver. (Note that the blood supply to the vascular compartment of the liver-draining lymph node is in the form of Equation (S.3), analogously to other lymph nodes.)

T cells that have not extravasated from the organ's vasculature return through major veins to the heart's right atrium and right ventricle, *RA&RV*, combined with the lymph node outflow:

$$\begin{aligned}
\frac{dN_{\text{RA\&RV}}}{dt} = V_{\text{RA\&RV}}\frac{dC_{\text{RA\&RV}}}{dt} &= -B_{\text{TOT}}C_{\text{RA\&RV}} \\
&+ \sum_{o\notin\{\text{mesentery}\}} \left(1-e_o\right)B_oC_o + \sum_{o\notin\{\text{spleen,PC}\}} \left(1-e_{\text{LN}_o}\right)B_{\text{LN}_o}C_{\text{LN}_o} \\
&+ \left(e_{\text{spleen}}\mu_{\text{spleen}}B_{\text{spleen}} + \sum_{o\in\{\text{mesentery}\}}\left(1-e_o\right)B_o\right)\left(1-e_{\text{liver}}\right)C_{\text{liver}} \\
&+ \sum_{\substack{o\notin\{\text{spleen,PC,}\\ \text{heart,lung}\}}} \left(e_{\text{LN}_o}\mu_{\text{LN}_o}B_{\text{LN}_o} + \mu_{\text{LN}_o}e_o\mu_oB_o\right)\tilde{C}_{\text{LN}_o} \\
&+ \left(e_{\text{LN}_{\text{med}}}\mu_{\text{LN}_{\text{med}}}B_{\text{LN}_{\text{med}}} + \mu_{\text{LN}_{\text{med}}}e_{\text{heart}}\mu_{\text{heart}}B_{\text{heart}} + \mu_{\text{LN}_{\text{med}}}e_{\text{lung}}\mu_{\text{lung}}B_{\text{lung}}\right)\tilde{C}_{\text{LN}_{\text{med}}} \\
&+ \mu_{\text{LN}_{\text{liver}}}e_{\text{liver}}\mu_{\text{liver}}\tilde{C}_{\text{LN}_{\text{liver}}}\left(e_{\text{spleen}}\mu_{\text{spleen}}B_{\text{spleen}} + \sum_{o\in\{\text{mesentery}\}} B_o\left(1-e_o\right)\right),
\end{aligned} \tag{S.10}$$

where $B_{\text{TOT}} = \sum_{o\notin\{PC\}} B_o + \sum_{o\notin\{PC,spleen\}} B_{\text{LN}_o}$ is the cardiac output, and the mesentery comprises of: the stomach, spleen, pancreas, small and large intestines. Further, each line respectively refers to: (i) the flow of T cells out of the heart's right atrium and right ventricle, (ii) the return of T cells from the non-mesenteric organs, (iii) the return of T cells from the mesentery via the liver, (iv) the return of T cells from the lymph nodes, including mediastinal (v) and liver-draining lymph nodes (vi).

T cells flow from the right atrium and right ventricle into the pulmonary circuit (PC), where the blood flow is equal to the cardiac output $B_{\text{TOT}} = \sum_{o\notin\{\text{PC}\}} B_o + \sum_{o\notin\{\text{PC, spleen}\}} B_{\text{LN}_o}$:

$$\frac{dN_{\text{PC}}}{dt} = B_{\text{TOT}}\left(C_{\text{RA\&RV}} - C_{\text{PC}}\right), \tag{S.11}$$

$$\frac{d\tilde{N}_{\text{PC}}}{dt} = e_{\text{PC}}B_{\text{TOT}}\left(C_{\text{PC}} - \mu_{\text{PC}}\tilde{C}_{\text{PC}}\right). \tag{S.12}$$

Note that the pulmonary circuit lacks lymphatics and the interstitial sub-compartment represents the 'trapped' state (functionally equivalent to interstitial space) [1, 2]. Finally, T cells return from the pulmonary circuit to the left atrium and left ventricle of the heart:

$$\frac{dN_{\text{LA\&LV}}}{dt} = (1-e_{\text{PC}})B_{\text{TOT}}C_{\text{PC}} + e_{\text{PC}}\mu_{\text{PC}}B_{\text{TOT}}\tilde{C}_{\text{PC}} - B_{\text{TOT}}C_{\text{LA\&LV}}, \tag{S.13}$$

where the last term describes T cells flowing through aorta and major arteries towards vasculature compartments, the subsequent downstream impact of which is captured in Equation (S.1).

2 Blood flow changes in the systemic circulation post-splenectomy

Given that a body tends to maintain the same cardiac output following splenectomy [3], a fraction of blood volume received by an organ, and, as a result, the blood flow through an organ, is expected to increase in the absence of the spleen. Hence, we seek to quantify the change in the blood flow of an organ post-splenectomy for the lymphocyte trafficking framework.

To proceed, we consider a circulation model based on the fundamental assumption of proportionality between the pressure drop across the arterioles, capillary bed and venules associated with an organ, o, and the blood flow through the organ, o, that is

$$\Delta P = R_o B_o, \tag{S.14}$$

where ΔP is the pressure drop, B_o is the blood flow through the organ, o, (defined to include the associated venules and arterioles in this context) and R_o is the resistance of the organ, o. This proportionality stems from the linear relationship between pressure drop and blood flow inherent in the Poiseuille flow through the smaller arterial and venule vessels, and also in vessel models of the capillary beds [4, 5] or more recent homogenised Darcy models of the capillary bed [6]. Validation of this model does require the additional physics of the Fahraeus effect and the Fahraeus-Lindqvist effect, which summarise a complex relation between vessel size, haematocrit and effective blood viscosity, as well as the phase-separation effect, encapsulating the redistribution of haematocrit at vessel bifurcations [5]. However, such physics governs the complex relation between the network geometry of an organ and its resistance to blood flow, R_o, rather than violating the proportionality between pressure drop and blood flow in the microcirculation. An important caveat in the use of Equation (S.14) concerns the level of vasodilation, which changes regionally and temporally, for example, with exercise or after a meal, and is anticipated to substantially alter organ resistance simply by the fact that resistance to flow is exceptionally sensitive to vessel dilation, scaling with the fourth power of the reciprocal of vessel diameter for simple fluids such as water. Hence, it is implicitly assumed below in comparisons of pre- and post- splenectomy blood flows that the body is in the same state of vasodilation. We also note that different organs will have different circulatory routes from, and back to, the heart and thus will experience different pressure drops due to the different circuit lengths of the feeding large arteries and draining large veins completing the cardiovascular circuit. However, the pressure drop across large arteries and large veins is subdominant compared to the arterioles, capillary beds and venules of the circulatory system (see Figure S.1) [7] and thus organ-specific variation in this subdominant contribution to the pressure drop across the cardiovascular circuit is also neglected below.

With these modelling assumptions in place, let the pressure difference between the large arteries and large veins with the spleen present (without the spleen) be denoted by ΔP (ΔP^*) and the blood flow through an organ, o, other than the spleen, liver and mesentery, when the spleen is present (without the spleen) be denoted by B_o (B_o^*). Then we have

$$R_o = \frac{\Delta P}{B_o} = \frac{\Delta P^*}{B_o^*}, \tag{S.15}$$

with analogous relations for the spleen, mesenteric organs and liver once the topology of the circulation is accommodated, as summarised in Figure S.2.

In particular, an organ, o, in the model can be thought of as a "resistor", where the blood flow, pressure drop and vascular resistance correspond to the current flowing through a resistor, the voltage across a resistor and its resistance. Hence, we can construct an electrical circuit (composed of resistors, battery and wires connecting all the components), which corresponds to the model compartments, arterial-venous pressure drop and major blood vessels connecting the organs. Such a circuit is shown in Figure S.2 (upper plane), where the mesenteric "circulation" is shown explicitly in the lower plane, and resistors corresponding to the remaining organs are represented in a simplified manner for clarity. The liver receives blood both from the hepatic artery (direct blood supply to the liver) and the hepatic portal vein, which drains from the mesenteric organs: the spleen, stomach, pancreas, small and large intestines. However, hepatic venous pressure gradient (HVPG), the pressure drop between the hepatic portal vein and the hepatic vein, is between 1 – 5 mmHg [8]. As a result, the contribution of the hepatic portal vein to the pressure drop across the cardiovascular circuit can be neglected, when compared to the dominant pressure drop across arterioles and venules associated with each mesenteric organ. Under such assumptions, an electrical circuit representing the circulation model simplifies to the circuit presented in Figure S.2 (lower plot).

To quantify the change in the blood flow through an organ o post-splenectomy, B_o^*, we consider the resistance of the circuit shown in Figure S.2 with the resistor R_{spleen} present and removed from the circuit. We make two assumptions: (i) the cardiac output remains constant after splenectomy ($B_{\text{TOT}} = B_{\text{TOT}}^*$), and (ii) splenectomy does not affect the vascular resistance of each organ ($R_o = R_o^*$).

By flow conservation, analogous to Kirchhoff's law in electric circuits, the cardiac output pre- and post splenectomy, B_{TOT} and B_{TOT}^*, can be expressed as a sum of the total blood flow through the spleen, B_{spleen}, and the remaining organs:

$$B_{\text{TOT}} = B_{\text{spleen}} + \sum_{\substack{o \notin \{\text{PC}, \\ \text{spleen}\}}} B_o = \sum_{\substack{o \notin \{\text{PC}, \\ \text{spleen}\}}} B_o^* = B_{\text{TOT}}^*. \tag{S.16}$$

Using Equation (S.14):

$$\frac{\Delta P}{R_{\text{spleen}}} + \sum_{\substack{o \notin \{\text{PC}, \\ \text{spleen}\}}} \frac{\Delta P}{R_o} = \sum_{\substack{o \notin \{\text{PC}, \\ \text{spleen}\}}} \frac{\Delta P^*}{R_o}, \tag{S.17}$$

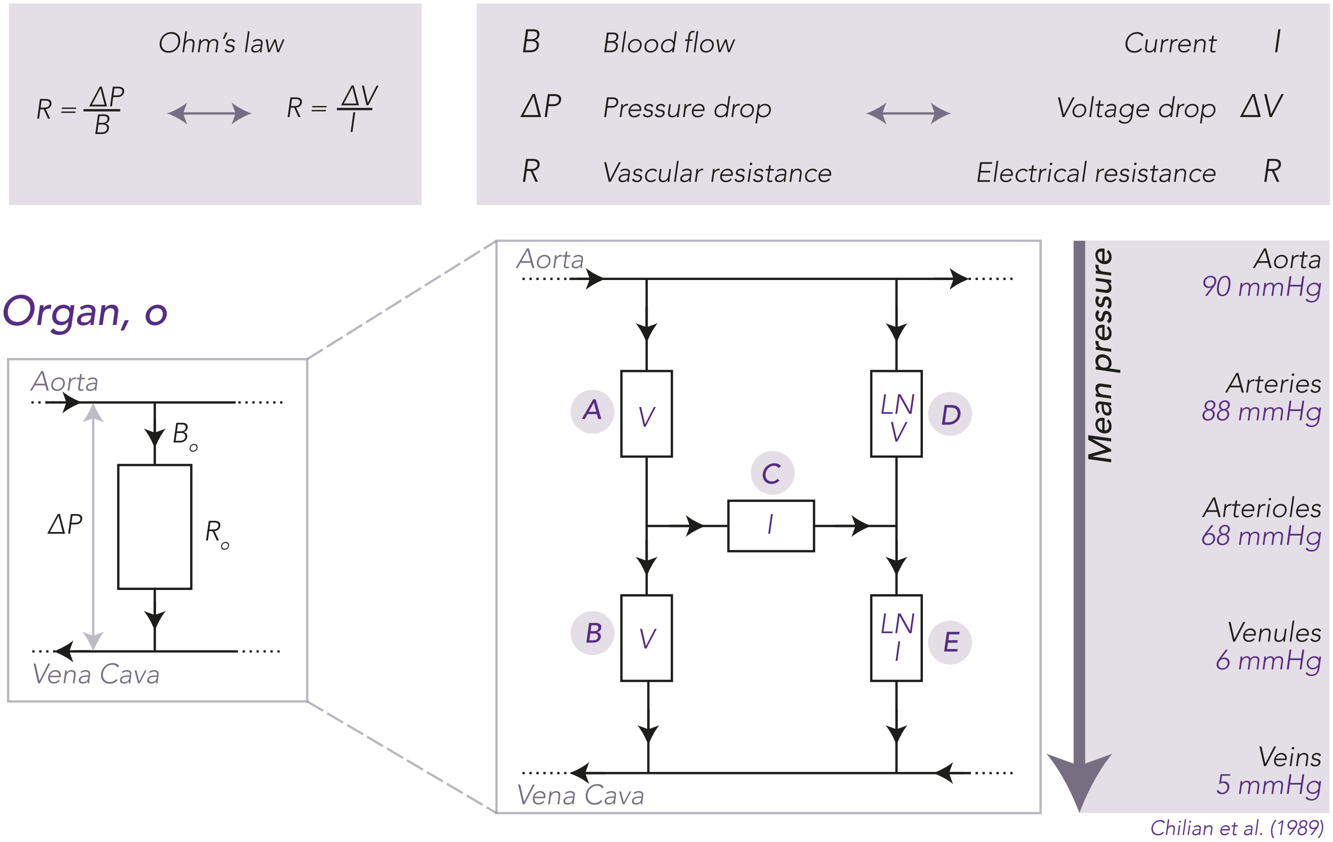

Figure S.1. The representation of organ, o, in the circulation model as a part of a Kirchhoff's circuit, with Kirchhoff's law of charge conservation, and thus a balance of currents, at nodes of the electric circuit analogous to a balance of flows at nodes in the circulation flow circuit. Continuing the analogy, the blood flow B_o, pressure drop ΔP and vascular resistance R_o, an organ o can be thought of as an Ohm's law resistor, where the blood flow, pressure drop and vascular resistance correspond to the current flowing through the "resistor", the voltage across the "resistor" and its resistance (left). In general, each organ will experience different pressure drops across different points in the circulation due to the different circulatory lengths and lymphatic drainage — from aorta and large arteries, between arterioles and venules (across capillary bed), to veins and vena cava (right). However, the pressure drops across the aorta and arteries (2 mmHg [7]), across arteries and arterioles (20 mmHg [7]), and across venules and veins (1 mmHg [7]) are small compared to the pressure drop from arterioles to venules (62 mmHg [7])and are, therefore, neglected. As a result, resistance associated with each organ represents the contributions from the vascular resistance of the organ before (A) and after (B) lymphatic drainage, the vascular resistance across the interstitium of the organ (C), the vascular resistance of the organ-draining lymph nodes (D) and the vascular resistance across the interstitium of the organ-draining lymph nodes (E), though this level of resolved detail is not required in the modelling presented here.

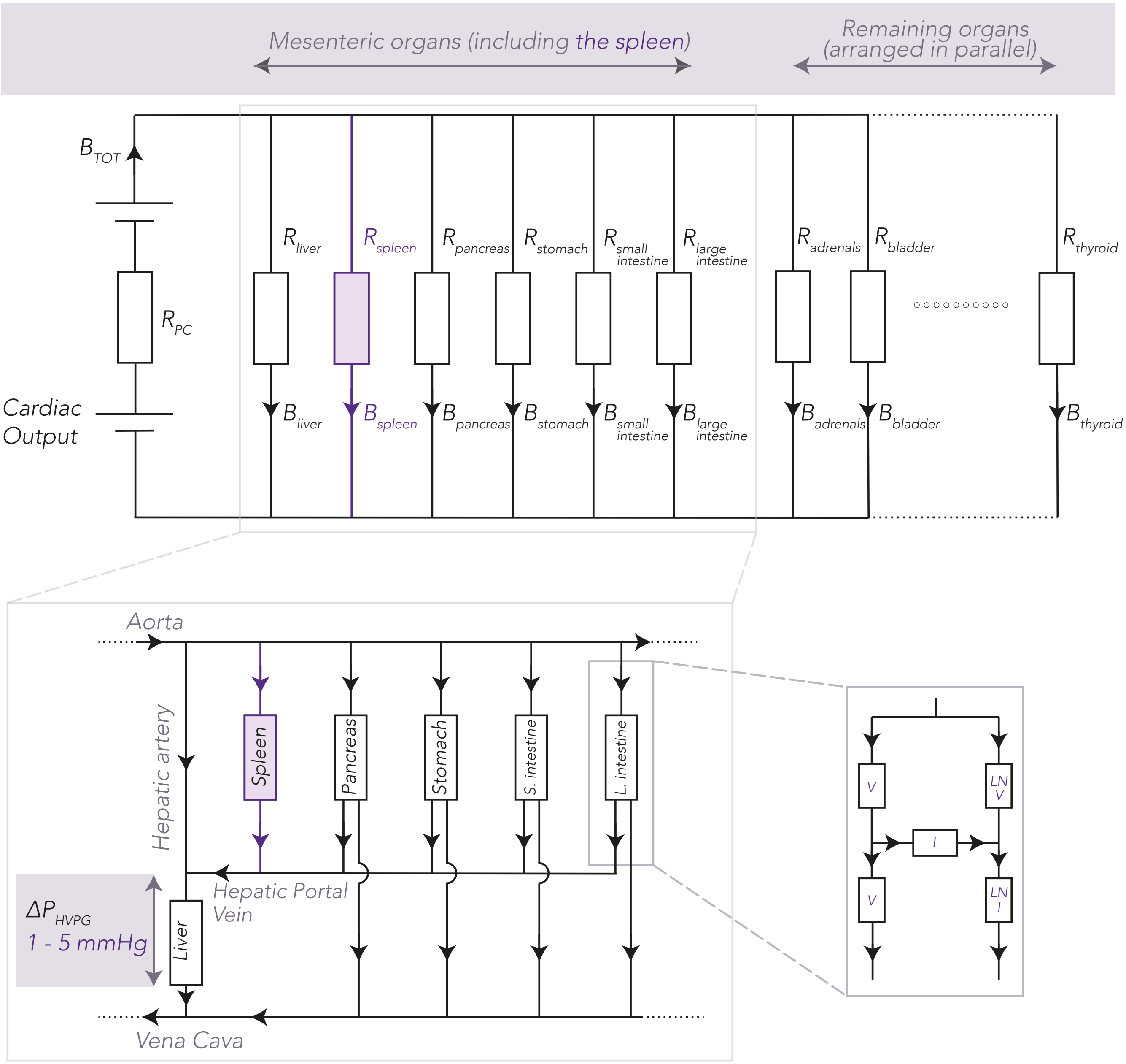

Figure S.2. The ODE model represented as a Kirchhoff circuit with Kirchhoff's law of charge conservation, and thus a balance of currents, at nodes of the electric circuit analogous to a balance of flows at nodes in the circulation flow circuit (upper). The liver receives blood both from the hepatic artery (direct blood supply to the liver) and the hepatic portal vein, which drains from the mesenteric organs: the spleen, stomach, pancreas, small and large intestines. However, since hepatic venous pressure drop (*HVPD*) is comparatively small compared to the pressure drop across arterioles and venules of any mesenteric organ, the contribution of the hepatic portal vein to the balance between fluxes and pressures is small, resulting in the modelling approximation of the circuit shown on the lower left (see Section 2). Note that the spleen lacks lymphatic drainage and that resistance associated with each organ (lower right) represents the contributions from the vascular resistance of the organ before and after lymphatic drainage, the vascular resistance across the interstitium of the organ, the vascular resistance of the organ-draining lymph nodes and the vascular resistance across the interstitium of the organ-draining lymph nodes, as indicated in Figure S.1, though this level of detail is not required in the modelling presented here.

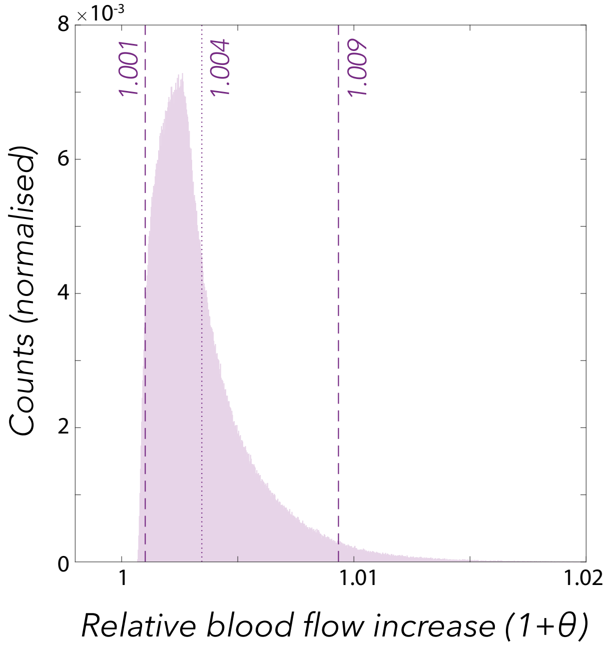

Figure S.3. Distribution of the relative blood flow increase, $1+\theta$, for 1,000,000 randomly sampled blood flow parameters from uniform distributions. Uniformly distributed blood flow values were drawn randomly for each organ in the ODE model, assuming the variability ranges as given in Table 2. The parameter θ was evaluated for each sampled set of parameters (Equation (S.20)) and the resulting distribution was plotted as a histograms with 1000 bins. Dotted and dashed lines correspond to the mean and the 95% confidence interval of the plotted distribution.

and hence:

$$\frac{\Delta P^*}{\Delta P} = 1 + \frac{\sum_{o \notin \{\text{spleen, PC}\}} R_o}{R_{\text{spleen}}} = 1 + \frac{B_{\text{spleen}}}{\sum_{o \notin \{\text{spleen, PC}\}} B_o} = \frac{B_{\text{TOT}}}{B_{\text{TOT}} - B_{\text{spleen}}}. \tag{S.18}$$

Using Equation (S.15), the blood flow through the organ o post-splenectomy can be expressed as:

$$\frac{B_o^*}{\Delta P^*} = \frac{B_o}{\Delta P}, \tag{S.19}$$

and substituting Equation (S.18) leads to the sought result:

$$B_o^* = \frac{B_{\text{TOT}}}{B_{\text{TOT}} - B_{\text{spleen}}} B_o = \left(1 + \frac{B_{\text{spleen}}}{B_{\text{TOT}} - B_{\text{spleen}}}\right) B_o = (1+\theta) B_o, \tag{S.20}$$

where θ describes the fractional increase in the blood flow through the organ o, in terms of blood flows measured in the presence of the spleen.

Blood flow parameter values were randomly sampled 1,000,000 times for each organ compartment from uniform probability distributions, defined by the variability ranges in Table 2. The relative blood flow increase, $1+\theta$, was evaluated for each sampled parameter set, and convergence was confirmed as noted in Section *In silico splenectomy - simulation* of the Main Text, with the resulting distribution plotted in Figure S.3. The distribution was characterised by a mean of 1.004 and 95% confidence interval 1.001 – 1.009, indicating that splenectomy does not increase blood flow through the remaining organs considerably within the framework.

3 *In silico* splenectomy — model reduction

Trivially neglecting initial transit dynamics, the ratio of post- to pre-splenectomy cell concentration in any compartment can be derived analytically by considering Equations (S.1) – (S.10) in steady state. Denote the pre-splenectomy cell concentration in organ o as C_o and the post-splenectomy cell concentration as C_o^*. The steady state of Equation (S.1) implies $C_o = C_{\text{LA\&LV}}$ and $C_o^* = C_{\text{LA\&LV}}^*$ for all organs $o \notin \{\text{liver, PC}\}$, meaning the steady-state cell concentration is equal to the cell concentration in the

left atrium and ventricle of the heart, $C_{\text{LA\&LV}}$ pre-splenectomy and $C^*_{\text{LA\&LV}}$ post-splenectomy for all organs, except the liver. We can therefore define a dimensionless constant κ such that:

$$C^*_o = \kappa C_o \text{ and } C^*_{\text{LA\&LV}} = \kappa C_{\text{LA\&LV}}, \tag{S.21}$$

and an expression for κ will be derived below. Similarly, by considering Equation (S.2) the steady-state cell concentration in the interstitium of organ, o, can be related to the steady-state concentration in the corresponding vasculature compartment: $\mu_o \tilde{C}_o = C_o$ (pre-splenectomy) and $\mu_o \tilde{C}^*_o = C^*_o$ (post-splenectomy). The interstitial cell concentrations pre- and post-splenectomy in organ, o, are related by the same constant κ analogously to Equation (S.21):

$$\tilde{C}^*_o = \kappa \tilde{C}_o. \tag{S.22}$$

Equations (S.21) and (S.22) imply that ratios of post- to pre-splenectomy cell concentration, and consequently cell numbers, are equal for both the vasculature and interstitial compartments of organ, o, and the ratios are equal for all organs. The exception is the liver, for which the equations are modified to capture the anatomy of hepatic blood and lymph circulation. By considering Equation (S.8) in steady state and substituting $C_{\text{LA\&LV}} = C_o$, $\tilde{C}_{\text{spleen}} = \frac{1}{\mu_{\text{spleen}}} C_{\text{spleen}} = \frac{1}{\mu_{\text{spleen}}} C_o$, the steady-state cell concentration C_{liver} can be solved for, resulting in:

$$\begin{aligned} C_{\text{liver}} &= \frac{B_{\text{liver}} + e_{\text{spleen}} B_{\text{spleen}} + \sum_{o \in \{\text{mesentery}\}} B_o (1 - e_o)}{B_{\text{liver}} + e_{\text{spleen}} \mu_{\text{spleen}} B_{\text{spleen}} + \sum_{o \in \{\text{mesentery}\}} B_o (1 - e_o)} C_o \\ &= \left(1 + \frac{e_{\text{spleen}} B_{\text{spleen}} (1 - \mu_{\text{spleen}})}{B_{\text{liver}} + e_{\text{spleen}} \mu_{\text{spleen}} B_{\text{spleen}} + \sum_{o \in \{\text{mesentery}\}} B_o (1 - e_o)} \right) C_o \\ &= \lambda C_o, \end{aligned} \tag{S.23}$$

where mesentery refers to the spleen, small and large intestines, stomach and pancreas and the variable λ has been introduced to simplify the notation. Equation (S.23) implies that the steady-state cell concentration in the liver vasculature is larger than in the remaining organs. Note that post-splenectomy $\lambda^* = 1$, since $B_{\text{spleen}} = 0$ and Equation (S.23) simplifies to:

$$C^*_{\text{liver}} = C^*_o, \tag{S.24}$$

indicating the cell concentration in the liver's vasculature post-splenectomy is the same as in the remaining organs. The ratio of post- to pre-splenectomy cell concentration in the liver vasculature is, therefore:

$$\frac{C^*_{\text{liver}}}{C_{\text{liver}}} = \frac{\kappa}{\left(1 + \frac{e_{\text{spleen}} B_{\text{spleen}} (1 - \mu_{\text{spleen}})}{B_{\text{liver}} + e_{\text{spleen}} \mu_{\text{spleen}} B_{\text{spleen}} + \sum_{o \in \{\text{mesentery}\}} B_o (1 - e_o)} \right)} = \frac{\kappa}{\lambda}. \tag{S.25}$$

Since $\lambda > 1$, the expected post-to pre-splenectomy cell concentration ratio is predicted to be lower than for the remaining compartments. Analogously, considering the steady state of Equation (S.8) and substituting $C_{\text{liver}} = \lambda C_o$ (Equation (S.23)), the steady-state cell concentration in the liver interstitium, $\tilde{C}_{\text{liver}}$, can be expressed as:

$$\tilde{C}_{\text{liver}} = \frac{C_{\text{liver}}}{\mu_{\text{liver}}} = \frac{\lambda}{\mu_{\text{liver}}} C_o. \tag{S.26}$$

Since $\lambda^* = 1$ in the absence of the spleen, the ratio of post- to pre-splenectomy cell concentration in the liver interstitium is given by:

$$\frac{\tilde{C}^*_{\text{liver}}}{\tilde{C}_{\text{liver}}} = \frac{C^*_o}{\lambda C_o} = \frac{\kappa}{\lambda}, \tag{S.27}$$

resulting in equal post- to pre-splenectomy ratios in both the vasculature and the interstitial liver compartments.

The post- to pre-splenectomy cell concentration ratio for organ, o, -draining lymph nodes can be derived in the same manner. Note that the lymph node blood supply compartment follows the same scaling as vasculature of organ, o, with:

$$C^*_{\text{LN}_o} = \kappa C_{\text{LN}_o}, \tag{S.28}$$

which follows from Equation (S.3). Consider Equation (S.4) for the interstitium compartment of organ, o, -draining lymph nodes in steady state. Substituting the known steady-state cell concentration relations ($C_{\text{LN}_o} = C_o$ and $\tilde{C}_o = \frac{1}{\mu_o} C_o$) and solving for $\tilde{C}_{\text{LN}_o}$ gives:

$$\tilde{C}_{\text{LN}_o} = \frac{e_{\text{LN}_o} B_{\text{LN}_o} + e_o B_o}{e_{\text{LN}_o} B_{\text{LN}_o} + e_o \mu_o B_o} \frac{C_o}{\mu_{\text{LN}_o}}. \tag{S.29}$$

Similarly, the steady-state cell concentration post-splenectomy, $\tilde{C}^*_{\mathrm{LN}_o}$, is given by:

$$\tilde{C}^*_{\mathrm{LN}_o} = \frac{e_{\mathrm{LN}_o} B^*_{\mathrm{LN}_o} + e_o B^*_o}{e_{\mathrm{LN}_o} B^*_{\mathrm{LN}_o} + e_o \mu_o B^*_o} \frac{C^*_o}{\mu_{\mathrm{LN}_o}} = \frac{e_{\mathrm{LN}_o} B_{\mathrm{LN}_o} + e_o B_o}{e_{\mathrm{LN}_o} B_{\mathrm{LN}_o} + e_o \mu_o B_o} \frac{C^*_o}{\mu_{\mathrm{LN}_o}}, \tag{S.30}$$

where $C^*_{\mathrm{LN}_o} = C^*_o$ and $\tilde{C}^*_o = \frac{1}{\mu_o} C^*_o$ were substituted and B^*_o denotes the blood flow post-splenectomy. Since all pre-splenectomy blood flows are scaled by the same factor $1+\theta$ (Equation (S.20)), the post- to pre-splenectomy cell concentration ratio is simply given by:

$$\frac{\tilde{C}^*_{\mathrm{LN}_o}}{\tilde{C}_{\mathrm{LN}_o}} = \frac{C^*_o}{C_o} = \kappa, \tag{S.31}$$

where κ is the post- to pre-splenectomy cell concentration ratio for all compartments, except the liver. Note that Equation (S.31) also holds for mediastinal lymph nodes, which drain both the heart and lung blood supply compartments. The ratio of the steady-state cell concentration in the mediastinal lymph node compartment post-splenectomy:

$$\begin{aligned} \tilde{C}^*_{\mathrm{LN}_{\mathrm{med}}} &= \frac{e_{\mathrm{LN}_{\mathrm{med}}} B^*_{\mathrm{LN}_{\mathrm{med}}} + e_{\mathrm{heart}} B^*_{\mathrm{heart}} + e_{\mathrm{lung}} B^*_{\mathrm{lung}}}{e_{\mathrm{LN}_{\mathrm{med}}} B^*_{\mathrm{LN}_{\mathrm{med}}} + e_{\mathrm{heart}} \mu_{\mathrm{heart}} B^*_{\mathrm{heart}} + e_{\mathrm{lung}} \mu_{\mathrm{lung}} B^*_{\mathrm{lung}}} \frac{C^*_o}{\mu_{\mathrm{LN}_{\mathrm{med}}}} \\ &= \frac{e_{\mathrm{LN}_{\mathrm{med}}} B_{\mathrm{LN}_{\mathrm{med}}} + e_{\mathrm{heart}} B_{\mathrm{heart}} + e_{\mathrm{lung}} B_{\mathrm{lung}}}{e_{\mathrm{LN}_{\mathrm{med}}} B_{\mathrm{LN}_{\mathrm{med}}} + e_{\mathrm{heart}} \mu_{\mathrm{heart}} B_{\mathrm{heart}} + e_{\mathrm{lung}} \mu_{\mathrm{lung}} B_{\mathrm{lung}}} \frac{C^*_o}{\mu_{\mathrm{LN}_{\mathrm{med}}}}, \end{aligned} \tag{S.32}$$

and pre-splenectomy:

$$\tilde{C}_{\mathrm{LN}_{\mathrm{med}}} = \frac{e_{\mathrm{LN}_{\mathrm{med}}} B_{\mathrm{LN}_{\mathrm{med}}} + e_{\mathrm{heart}} B_{\mathrm{heart}} + e_{\mathrm{lung}} B_{\mathrm{lung}}}{e_{\mathrm{LN}_{\mathrm{med}}} B_{\mathrm{LN}_{\mathrm{med}}} + e_{\mathrm{heart}} \mu_{\mathrm{heart}} B_{\mathrm{heart}} + e_{\mathrm{lung}} \mu_{\mathrm{lung}} B_{\mathrm{lung}}} \frac{C_o}{\mu_{\mathrm{LN}_{\mathrm{med}}}} \tag{S.33}$$

yields the same post- to pre-splenectomy cell concentration ratio, κ:

$$\frac{\tilde{C}^*_{\mathrm{LN}_{\mathrm{med}}}}{\tilde{C}_{\mathrm{LN}_{\mathrm{med}}}} = \frac{C^*_o}{C_o} = \kappa. \tag{S.34}$$

Also note that the post- to pre-splenectomy ratio remains the same independently of the number of drained organs, with the exception of the liver. The post- to pre-splenectomy cell concentration ratio in lymph nodes that do not drain from the liver are, therefore, equal to the ratio for all other organs, except the liver.

Due to the anatomy of hepatic circulation, the liver-draining lymph nodes differ from the remaining lymph nodes. Consider Equation (S.9) in steady state:

$$\begin{aligned} \frac{d\tilde{N}_{\mathrm{LN}_{\mathrm{liver}}}}{dt} &= \tilde{V}_{\mathrm{LN}_{\mathrm{liver}}} \frac{d\tilde{C}_{\mathrm{LN}_{\mathrm{liver}}}}{dt} = e_{\mathrm{LN}_{\mathrm{liver}}} B_{\mathrm{LN}_{\mathrm{liver}}} \left(C_{\mathrm{LN}_{\mathrm{liver}}} - \mu_{\mathrm{LN}_{\mathrm{liver}}} \tilde{C}_{\mathrm{LN}_{\mathrm{liver}}} \right) + e_{\mathrm{liver}} \mu_{\mathrm{liver}} B_{\mathrm{liver}} \left(\tilde{C}_{\mathrm{liver}} - \mu_{\mathrm{LN}_{\mathrm{liver}}} \tilde{C}_{\mathrm{LN}_{\mathrm{liver}}} \right) \\ &\quad + e_{\mathrm{liver}} \mu_{\mathrm{liver}} \left(e_{\mathrm{spleen}} \mu_{\mathrm{spleen}} B_{\mathrm{spleen}} + \sum_{o \in \{\mathrm{mesentery}\}} B_o (1 - e_o) \right) \left(\tilde{C}_{\mathrm{liver}} - \mu_{\mathrm{LN}_{\mathrm{liver}}} \tilde{C}_{\mathrm{LN}_{\mathrm{liver}}} \right) = 0, \end{aligned}$$

Recall that pre-splenectomy, in steady state $C_{\mathrm{LN}_{\mathrm{liver}}} = C_o$ and $\tilde{C}_{\mathrm{liver}} = \frac{\lambda}{\mu_{\mathrm{liver}}} C_o$, where λ is given by Equation (S.23). Substituting known steady-state cell concentrations and solving for $\tilde{C}_{\mathrm{LN}_{\mathrm{liver}}}$ in steady state:

$$\begin{aligned} \tilde{C}_{\mathrm{LN}_{\mathrm{liver}}} &= \frac{e_{\mathrm{LN}_{\mathrm{liver}}} B_{\mathrm{LN}_{\mathrm{liver}}} + \lambda e_{\mathrm{liver}} \left(B_{\mathrm{liver}} + e_{\mathrm{spleen}} \mu_{\mathrm{spleen}} B_{\mathrm{spleen}} + \sum_m B_m (1 - e_m) \right)}{e_{\mathrm{LN}_{\mathrm{liver}}} B_{\mathrm{LN}_{\mathrm{liver}}} + e_{\mathrm{liver}} \mu_{\mathrm{liver}} \left(B_{\mathrm{liver}} + e_{\mathrm{spleen}} \mu_{\mathrm{spleen}} B_{\mathrm{spleen}} + \sum_m B_m (1 - e_m) \right)} \frac{C_o}{\mu_{\mathrm{LN}_{\mathrm{liver}}}} \\ &= \sigma \frac{C_o}{\mu_{\mathrm{LN}_{\mathrm{liver}}}}, \end{aligned} \tag{S.35}$$

which defines σ, where summation over m runs over mesenteric organs (the spleen, stomach, small and large intestine, and pancreas). Similarly, for the post-splenectomy steady-state cell concentration $\tilde{C}^*_{\mathrm{LN}_{\mathrm{liver}}}$:

$$\begin{aligned} \tilde{C}^*_{\mathrm{LN}_{\mathrm{liver}}} &= \frac{e_{\mathrm{LN}_{\mathrm{liver}}} B_{\mathrm{LN}_{\mathrm{liver}}} + e_{\mathrm{liver}} \left(B_{\mathrm{liver}} + \sum_{m^*} B_{m^*} (1 - e_{m^*}) \right)}{e_{\mathrm{LN}_{\mathrm{liver}}} B_{\mathrm{LN}_{\mathrm{liver}}} + e_{\mathrm{liver}} \mu_{\mathrm{liver}} \left(B_{\mathrm{liver}} + \sum_{m^*} B_{m^*} (1 - e_{m^*}) \right)} \frac{C_o}{\mu_{\mathrm{LN}_{\mathrm{liver}}}} \\ &= \sigma^* \frac{C^*_o}{\mu_{\mathrm{LN}_{\mathrm{liver}}}}, \end{aligned} \tag{S.36}$$

which defines σ^*, where summation over m^* runs over mesenteric organs post-splenectomy (the stomach, small and large intestine, and pancreas). The post- to pre-splenectomy cell concentration ratio can be then expressed as

$$\frac{\tilde{C}^*_{\mathrm{LN_{liver}}}}{\tilde{C}_{\mathrm{LN_{liver}}}} = \frac{\sigma^*}{\sigma}\frac{C^*_o}{C_o} = \frac{\sigma^*\kappa}{\sigma}, \tag{S.37}$$

where the parameter groupings σ and σ^* were derived above.

Finally, it can be shown that the post- to pre-splenectomy cell concentration for the three remaining compartments, the pulmonary circuit (PC), the right (RA&RV) and left heart chambers (LA&LV), is equal to κ. By considering Equation (S.10) in steady state pre-splenectomy (post-splenectomy) we have:

$$C_{\mathrm{PC}} = C_{\mathrm{RA\&RV}} \quad C^*_{\mathrm{PC}} = C^*_{\mathrm{RA\&RV}}, \tag{S.38}$$

and similarly Equation (S.12) gives:

$$\tilde{C}_{\mathrm{PC}} = \frac{1}{\mu_{\mathrm{PC}}} C_{\mathrm{PC}} \quad \left(\tilde{C}^*_{\mathrm{PC}} = \frac{1}{\mu_{\mathrm{PC}}} C^*_{\mathrm{PC}}\right). \tag{S.39}$$

Substituting the above relations into Equation (S.13) in steady state yields:

$$C_{\mathrm{LA\&LV}} = C_{\mathrm{PC}} = C_{\mathrm{RA\&RV}} \quad \left(C^*_{\mathrm{LA\&LV}} = C^*_{\mathrm{PC}} = C^*_{\mathrm{RA\&RV}}\right), \tag{S.40}$$

and, by Equation (S.21), the post- to pre-splenectomy cell concentration ratios in the pulmonary circuit (PC) and heart chambers compartments (RA&RV and LA&LV) are:

$$\frac{C^*_{\mathrm{LA\&LV}}}{C_{\mathrm{LA\&LV}}} = \frac{C^*_{\mathrm{RA\&RV}}}{C_{\mathrm{RA\&RV}}} = \frac{C^*_{\mathrm{PC}}}{C_{\mathrm{PC}}} = \frac{\tilde{C}^*_{\mathrm{PC}}}{\tilde{C}_{\mathrm{PC}}} = \kappa. \tag{S.41}$$

An expression for the post- to pre-splenectomy concentration ratio κ in organs other than the liver and liver-draining lymph nodes (Equation (S.21) and (S.22)) is derived from the constraint that the total number of cells in the system is conserved pre- and post-splenectomy: $N_{\mathrm{TOT}} = N^*_{\mathrm{TOT}}$. Expressing the constraint via a summation of the number of cells in each compartment in steady state:

$$\begin{aligned} N_{\mathrm{TOT}} &= \sum_o V_o C_o + \sum_{\substack{o \notin \{\mathrm{RA\&RV} \\ \mathrm{PC,LA\&LV}\}}} \tilde{V}_o \tilde{C}_o + \sum_{\substack{o' \notin \{\mathrm{spleen,RA\&RV,} \\ \mathrm{PC,LA\&LV}\}}} \left(V_{\mathrm{LN}_{o'}} C_{\mathrm{LN}_{o'}} + \tilde{V}_{\mathrm{LN}_{o'}} \tilde{C}_{\mathrm{LN}_{o'}}\right) \\ N^*_{\mathrm{TOT}} &= \sum_{o \notin \{\mathrm{spleen}\}} V_o C^*_o + \sum_{\substack{o \notin \{\mathrm{spleen,RA\&RV,} \\ \mathrm{PC,LA\&LV}\}}} \tilde{V}_o \tilde{C}^*_o + \sum_{\substack{o' \notin \{\mathrm{spleen,RA\&RV,} \\ \mathrm{PC,LA\&LV}\}}} \left(V_{\mathrm{LN}_{o'}} C^*_{\mathrm{LN}_{o'}} + \tilde{V}_{\mathrm{LN}_{o'}} \tilde{C}^*_{\mathrm{LN}_{o'}}\right). \end{aligned} \tag{S.42}$$

Substituting the derived expressions for the cell concentration pre- and post-splenectomy in the liver and lymph nodes compartments and one can write N_{TOT} in the form $N_{\mathrm{TOT}} = AC_o$, where

$$A = \sum_{o \notin \{\mathrm{liver}\}} V_o + \sum_{\substack{o \notin \{\mathrm{liver,RA} \\ \mathrm{\&RV,LA\&LV}\}}} \frac{\tilde{V}_o}{\mu_o} + \lambda\left(V_{\mathrm{liver}} + \frac{\tilde{V}_{\mathrm{liver}}}{\mu_{\mathrm{liver}}}\right) + \sum_{\substack{o' \notin \{\mathrm{spleen,RA\&RV,} \\ \mathrm{PC,LA\&LV,liver}\}}} \left(V_{\mathrm{LN}_{o'}} + \frac{\tilde{V}_{\mathrm{LN}_{o'}}}{\mu_{\mathrm{LN}_{o'}}}\right) + V_{\mathrm{LN_{liver}}} + \sigma \frac{\tilde{V}_{\mathrm{LN_{liver}}}}{\mu_{\mathrm{LN_{liver}}}} \tag{S.43}$$

using, for example, $C_{\mathrm{LN}_o} = C_o$ for all organs including liver, and $\tilde{C}_{\mathrm{LN}_o} = \frac{1}{\mu_{\mathrm{LN}_o}} C_o$ for all organs but the liver, where $\tilde{C}_{\mathrm{LN_{liver}}} = \frac{\sigma}{\mu_{\mathrm{LN_{liver}}}} C_o$. With analogous identities and algebra post-splenectomy we have $N^*_{\mathrm{TOT}} = BC^*_o$, where

$$B = \sum_{o \notin \{\mathrm{spleen}\}} V_o + \sum_{\substack{o \notin \{\mathrm{spleen,RA} \\ \mathrm{\&RV,LA\&LV}\}}} \frac{\tilde{V}_o}{\mu_o} + \sum_{\substack{o' \notin \{\mathrm{spleen,RA\&RV,} \\ \mathrm{PC,LA\&LV,liver}\}}} \left(V_{\mathrm{LN}_{o'}} + \frac{\tilde{V}_{\mathrm{LN}_{o'}}}{\mu_{\mathrm{LN}_{o'}}}\right) + V_{\mathrm{LN_{liver}}} + \sigma^* \frac{\tilde{V}_{\mathrm{LN_{liver}}}}{\mu_{\mathrm{LN_{liver}}}}. \tag{S.44}$$

Hence with $N_{\mathrm{TOT}} = N^*_{\mathrm{TOT}}$, we have $AC_o = BC^*_o$ and thus:

$$\kappa = \frac{\sum\limits_{o \notin \{\mathrm{liver}\}} V_o + \sum\limits_{\substack{o \notin \{\mathrm{liver,RA} \\ \mathrm{\&RV,LA\&LV}\}}} \frac{\tilde{V}_o}{\mu_o} + \lambda\left(V_{\mathrm{liver}} + \frac{\tilde{V}_{\mathrm{liver}}}{\mu_{\mathrm{liver}}}\right) + \sum\limits_{\substack{o' \notin \{\mathrm{spleen,RA\&RV,} \\ \mathrm{PC,LA\&LV,liver}\}}} \left(V_{\mathrm{LN}_{o'}} + \frac{\tilde{V}_{\mathrm{LN}_{o'}}}{\mu_{\mathrm{LN}_{o'}}}\right) + V_{\mathrm{LN_{liver}}} + \sigma \frac{\tilde{V}_{\mathrm{LN_{liver}}}}{\mu_{\mathrm{LN_{liver}}}}}{\sum\limits_{o \notin \{\mathrm{spleen}\}} V_o + \sum\limits_{\substack{o \notin \{\mathrm{spleen,RA} \\ \mathrm{\&RV,LA\&LV}\}}} \frac{\tilde{V}_o}{\mu_o} + \sum\limits_{\substack{o' \notin \{\mathrm{spleen,RA\&RV,} \\ \mathrm{PC,LA\&LV,liver}\}}} \left(V_{\mathrm{LN}_{o'}} + \frac{\tilde{V}_{\mathrm{LN}_{o'}}}{\mu_{\mathrm{LN}_{o'}}}\right) + V_{\mathrm{LN_{liver}}} + \sigma^* \frac{\tilde{V}_{\mathrm{LN_{liver}}}}{\mu_{\mathrm{LN_{liver}}}}}. \tag{S.45}$$

Compartments	Pre-splenectomy	Post-splenectomy	Ratio
Liver	$C_{\text{liver}} = \lambda C_o$	$C^*_{\text{liver}} = C^*_o = \kappa C_o$	κ/λ
	$\tilde{C}_{\text{liver}} = \frac{\lambda}{\mu_{\text{liver}}} C_o$	$\tilde{C}^*_{\text{liver}} = \frac{1}{\mu_{\text{liver}}} C^*_o = \frac{\kappa}{\mu_{\text{liver}}} C_o$	κ/λ
Liver-draining lymph nodes	$C_{\text{LN}_{\text{liver}}} = C_o$	$C^*_{\text{LN}_{\text{liver}}} = C^*_o = \kappa C_o$	κ
	$\tilde{C}_{\text{LN}_{\text{liver}}} = \frac{\sigma}{\mu_{\text{LN}_{\text{liver}}}} C_o$	$\tilde{C}^*_{\text{LN}_{\text{liver}}} = \frac{\sigma^*}{\mu_{\text{LN}_{\text{liver}}}} C^*_o = \frac{\sigma^* \kappa}{\mu_{\text{LN}_{\text{liver}}}} C_o$	$\sigma^* \kappa/\sigma$
Remaining organs	C_o	$C^*_o = \kappa C_o$	κ
	$\tilde{C}_o = \frac{1}{\mu_o} C_o$	$\tilde{C}^*_o = \kappa \tilde{C}_o = \frac{\kappa}{\mu_o} C_o$	κ
Remaining lymph nodes	$C_{\text{LN}_o} = C_o$	$C^*_{\text{LN}_o} = C^*_o = \kappa C_o$	κ
	$\tilde{C}_{\text{LN}} = \frac{1}{\mu_{\text{LN}_o}} C_o$	$\tilde{C}^*_{\text{LN}_o} = \frac{1}{\mu_{\text{LN}_o}} C^*_o = \frac{\kappa}{\mu_{\text{LN}_o}} C_o$	κ

$$\kappa = \frac{\sum_{o \notin \{\text{liver}\}} V_o + \sum_{\substack{o \notin \{\text{liver,RA} \\ \text{\&RV,LA\&LV}\}}} \frac{\tilde{V}_o}{\mu_o} + \lambda \left(V_{\text{liver}} + \frac{\tilde{V}_{\text{liver}}}{\mu_{\text{liver}}} \right) + \sum_{\substack{o' \notin \{\text{spleen,RA\&RV,} \\ \text{PC,LA\&LV,liver}\}}} \left(V_{\text{LN}_{o'}} + \frac{\tilde{V}_{\text{LN}_{o'}}}{\mu_{\text{LN}_{o'}}} \right) + V_{\text{LN}_{\text{liver}}} + \sigma \frac{\tilde{V}_{\text{LN}_{\text{liver}}}}{\mu_{\text{LN}_{\text{liver}}}}}{\sum_{o \notin \{\text{spleen}\}} V_o + \sum_{\substack{o \notin \{\text{spleen,RA} \\ \text{\&RV,LA\&LV}\}}} \frac{\tilde{V}_o}{\mu_o} + \sum_{\substack{o' \notin \{\text{spleen,RA\&RV,} \\ \text{PC,LA\&LV,liver}\}}} \left(V_{\text{LN}_{o'}} + \frac{\tilde{V}_{\text{LN}_{o'}}}{\mu_{\text{LN}_{o'}}} \right) + V_{\text{LN}_{\text{liver}}} + \sigma^* \frac{\tilde{V}_{\text{LN}_{\text{liver}}}}{\mu_{\text{LN}_{\text{liver}}}}}, \text{ where:}$$

$$\lambda = 1 + \frac{e_{\text{spleen}} B_{\text{spleen}} (1 - \mu_{\text{spleen}})}{B_{\text{liver}} + e_{\text{spleen}} \mu_{\text{spleen}} B_{\text{spleen}} + \sum_m B_m (1 - e_m)}$$

$$\sigma = \frac{e_{\text{LN}_{\text{liver}}} B_{\text{LN}_{\text{liver}}} + \lambda e_{\text{liver}} (B_{\text{liver}} + e_{\text{spleen}} \mu_{\text{spleen}} B_{\text{spleen}} + \sum_m B_m (1 - e_m))}{e_{\text{LN}_{\text{liver}}} B_{\text{LN}_{\text{liver}}} + e_{\text{liver}} \mu_{\text{liver}} (B_{\text{liver}} + e_{\text{spleen}} \mu_{\text{spleen}} B_{\text{spleen}} + \sum_m B_m (1 - e_m))}$$

$$\sigma * = \frac{e_{\text{LN}_{\text{liver}}} B_{\text{LN}_{\text{liver}}} + e_{\text{liver}} (B_{\text{liver}} + \sum_{m^*} B_{m^*} (1 - e_{m^*}))}{e_{\text{LN}_{\text{liver}}} B_{\text{LN}_{\text{liver}}} + e_{\text{liver}} \mu_{\text{liver}} (B_{\text{liver}} + \sum_{m^*} B_{m^*} (1 - e_{m^*}))}$$

where $m \in$ {spleen, small intestine, large intestine, stomach, pancreas} and $m^* \in$ {small intestine, large intestine, stomach, pancreas}. Note that λ, σ and σ^* are independent of volumes.

Table S.1. Summary of the derived post- to pre-splenectomy cell concentration ratios in all model compartments.

A summary of derived ratios is shown in Table S.1.

Using the derived variables, two ratios of interest can be constructed: the post- to pre-splenectomy total T cell localisation to SLT ratio, $N^*_{\mathrm{SLT}}/N_{\mathrm{SLT}}$, and post- to pre-splenectomy net T cell concentration in SLT ratio, $C^*_{\mathrm{SLT}}/C_{\mathrm{SLT}}$. The total T cell localisation ratio is taken as a ratio of the total number of cells localising to all lymph nodes post-splenectomy, N^*_{SLT}:

$$
\begin{aligned}
N^*_{\mathrm{SLT}} &= \sum_{\substack{o\notin\{\text{spleen,RA\&RV,}\\ \text{PC,LA\&LV}\}}} \left(N^*_{\mathrm{LN}_o} + \tilde{N}^*_{\mathrm{LN}_o}\right) = \sum_{\substack{o\notin\{\text{spleen,RA\&RV,}\\ \text{PC,LA\&LV}\}}} \left(V_{\mathrm{LN}_o} C^*_{\mathrm{LN}_o} + \tilde{V}_{\mathrm{LN}_o} \tilde{C}^*_{\mathrm{LN}_o}\right) \\
&= \sum_{\substack{o\notin\{\text{spleen,RA\&RV,}\\ \text{PC,LA\&LV,liver}\}}} \left(V_{\mathrm{LN}_o} C^*_o + \tilde{V}_{\mathrm{LN}_o} \frac{C^*_o}{\mu_{\mathrm{LN}_o}}\right) + V_{\mathrm{LN}_{\mathrm{liver}}} C^*_o + \tilde{V}_{\mathrm{LN}_{\mathrm{liver}}} \frac{\sigma}{\mu_{\mathrm{LN}_{\mathrm{liver}}}} C^*_o \\
&= \sum_{\substack{o\notin\{\text{spleen,RA\&RV,}\\ \text{PC,LA\&LV, liver}\}}} \left(V_{\mathrm{LN}_o} \kappa C_o + \tilde{V}_{\mathrm{LN}_o} \frac{\kappa C_o}{\mu_{\mathrm{LN}_o}}\right) + V_{\mathrm{LN}_{\mathrm{liver}}} \kappa C_o + \tilde{V}_{\mathrm{LN}_{\mathrm{liver}}} \frac{\kappa\sigma^*}{\mu_{\mathrm{LN}_{\mathrm{liver}}}} C_o,
\end{aligned}
\tag{S.46}
$$

to the total number of T cells localising to all lymph nodes and the spleen pre-splenectomy,

$$
\begin{aligned}
N_{\mathrm{SLT}} &= N_{\mathrm{spleen}} + \tilde{N}_{\mathrm{spleen}} + \sum_{\substack{o\notin\{\text{spleen,RA\&RV,}\\ \text{PC,LA\&LV}\}}} \left(N_{\mathrm{LN}_o} + \tilde{N}_{\mathrm{LN}_o}\right) \\
&= V_{\mathrm{spleen}} C_{\mathrm{spleen}} + \tilde{V}_{\mathrm{spleen}} \tilde{C}_{\mathrm{spleen}} + \sum_{\substack{o\notin\{\text{spleen,RA\&RV,}\\ \text{PC,LA\&LV}\}}} \left(V_{\mathrm{LN}_o} C_{\mathrm{LN}_o} + \tilde{V}_{\mathrm{LN}_o} \tilde{C}_{\mathrm{LN}_o}\right) \\
&= V_{\mathrm{spleen}} C_{\mathrm{o}} + \tilde{V}_{\mathrm{spleen}} \frac{C_o}{\mu_{\mathrm{spleen}}} + \sum_{\substack{o\notin\{\text{spleen,RA\&RV,}\\ \text{PC,LA\&LV,liver}\}}} \left(V_{\mathrm{LN}_o} C_o + \tilde{V}_{\mathrm{LN}_o} \frac{C_o}{\mu_{\mathrm{LN}_o}}\right) \\
&+ V_{\mathrm{LN}_{\mathrm{liver}}} C_o + \tilde{V}_{\mathrm{LN}_{\mathrm{liver}}} \frac{\sigma C_o}{\mu_{\mathrm{LN}_{\mathrm{liver}}}}.
\end{aligned}
\tag{S.47}
$$

Hence the total SLT T cell localisation ratio is

$$
\frac{N^*_{\mathrm{SLT}}}{N_{\mathrm{SLT}}} = \kappa \frac{\sum_{\substack{o\notin\{\text{spleen,RA\&RV,}\\ \text{PC,LA\&LV,liver}\}}} \left(V_{\mathrm{LN}_o} + \tilde{V}_{\mathrm{LN}_o} \frac{1}{\mu_{\mathrm{LN}_o}}\right) + V_{\mathrm{LN}_{\mathrm{liver}}} + \tilde{V}_{\mathrm{LN}_{\mathrm{liver}}} \frac{\sigma^*}{\mu_{\mathrm{LN}_{\mathrm{liver}}}}}{V_{\mathrm{spleen}} + \tilde{V}_{\mathrm{spleen}} \frac{1}{\mu_{\mathrm{spleen}}} + \sum_{\substack{o\notin\{\text{spleen,RA\&RV,}\\ \text{PC,LA\&LV,liver}\}}} \left(V_{\mathrm{LN}_o} + \tilde{V}_{\mathrm{LN}_o} \frac{1}{\mu_{\mathrm{LN}_o}}\right) + V_{\mathrm{LN}_{\mathrm{liver}}} + \tilde{V}_{\mathrm{LN}_{\mathrm{liver}}} \frac{\sigma}{\mu_{\mathrm{LN}_{\mathrm{liver}}}}},
\tag{S.48}
$$

where κ, σ, σ^* are given by Equation (S.45), (S.35), (S.36), respectively. The net SLT T cell concentration ratio, $C^*_{\mathrm{SLT}}/C_{\mathrm{SLT}}$, can then be defined as the ratio of the total number of T cells in SLT per unit volume of SLT post- to pre-splenectomy:

$$
\frac{\bar{C}^*_{\mathrm{SLT}}}{\bar{C}_{\mathrm{SLT}}} = \frac{N^*_{\mathrm{SLT}}}{N_{\mathrm{SLT}}} \Bigg/ \frac{\sum_{\substack{o\notin\{\text{spleen,RA\&RV,}\\ \text{PC,LA\&LV}\}}} \tilde{V}_{\mathrm{LN}_o}}{\tilde{V}_{\mathrm{spleen}} + \sum_{\substack{o\notin\{\text{spleen,RA\&RV,}\\ \text{PC,LA\&LV}\}}} \tilde{V}_{\mathrm{LN}_o}}\,.
\tag{S.49}
$$

Assuming identical return parameters for all organ-draining lymph nodes, μ_{LN}, Equations (S.48) and (S.49) can be rewritten in the form used in the global sensitivity analysis eFAST

$$
\frac{N^*_{\mathrm{SLT}}}{N_{\mathrm{SLT}}} = \kappa \frac{V_{\mathrm{LN}_{\mathrm{TOT}}} + \frac{1}{\mu_{\mathrm{LN}}} \tilde{V}_{\mathrm{LN}_{\mathrm{TOT}}} \left(1 + (\sigma^* - 1) \frac{\tilde{V}_{\mathrm{liver}}}{\sum_{\substack{i\notin\{\text{spleen,PC,}\\ \text{RA\&RV,LA\&LV}\}}} \tilde{V}_i}\right)}{V_{\mathrm{spleen}} + \frac{1}{\mu_{\mathrm{spleen}}} \tilde{V}_{\mathrm{spleen}} + V_{\mathrm{LN}_{\mathrm{TOT}}} + \frac{1}{\mu_{\mathrm{LN}}} \tilde{V}_{\mathrm{LN}_{\mathrm{TOT}}} \left(1 + (\sigma - 1) \frac{\tilde{V}_{\mathrm{liver}}}{\sum_{\substack{i\notin\{\text{spleen,PC,}\\ \text{RA\&RV,LA\&LV}\}}} \tilde{V}_i}\right)},
\tag{S.50}
$$

$$
\frac{\bar{C}^*_{\mathrm{SLT}}}{\bar{C}_{\mathrm{SLT}}} = \frac{N^*_{\mathrm{SLT}}}{N_{\mathrm{SLT}}} \Bigg/ \frac{\tilde{V}_{\mathrm{LN}_{\mathrm{TOT}}}}{\tilde{V}_{\mathrm{LN}_{\mathrm{TOT}}} + \tilde{V}_{\mathrm{spleen}}}\,,
\tag{S.51}
$$

where $V_{\text{LN}_{\text{TOT}}}$ and $\tilde{V}_{\text{LN}_{\text{TOT}}}$ is the total blood and organ volume of lymph nodes, respectively. The derivation follows in a straightforward manner. For example, note the liver is excluded in the summation in the numerator of Equation (S.48). Reinstating the liver in this summation by adding the liver contribution to the summation and subtracting it from the remaining terms entails this numerator can be written as

$$\sum_{\substack{o\notin\{\text{spleen,RA\&RV,}\\ \text{PC,LA\&LV }\}}} \left(V_{\text{LN}_o} + \tilde{V}_{\text{LN}_o}\frac{1}{\mu_{\text{LN}}}\right) + (\sigma^* - 1)\frac{\tilde{V}_{\text{LN}_{\text{liver}}}}{\mu_{\text{LN}}} = V_{\text{LN}_{\text{TOT}}} + \frac{1}{\mu_{\text{LN}}}\tilde{V}_{\text{LN}_{\text{TOT}}} + (\sigma^* - 1)\frac{\tilde{V}_{\text{LN}_{\text{liver}}}}{\mu_{\text{LN}}} \tag{S.52}$$

$$= V_{\text{LN}_{\text{TOT}}} + \frac{1}{\mu_{\text{LN}}}\tilde{V}_{\text{LN}_{\text{TOT}}} + \frac{(\sigma^* - 1)}{\mu_{\text{LN}}}\tilde{V}_{\text{LN}_{\text{TOT}}} \frac{\tilde{V}_{\text{liver}}}{\sum_{\substack{i\notin\{\text{spleen,PC,}\\ \text{RA\&RV,LA\&LV}\}}} \tilde{V}_i},$$

where the final line arises by application of the partitioning of Equation (3) in the Main Text. Furthermore this final line gives the numerator of Equation (S.50) and the equivalence of the denominator in Equations (S.48) and (S.50) follows analogously.

4 Skeleton compartment parameterisation

The T cell trafficking model presented in the Main Text includes a 'Skeleton' compartment, which was parameterised to account for the contribution of both the bone marrow and bones. We have performed the analysis presented in the Main Text of the manuscript with the parameterisation of the skeleton compartment restricted to the bone marrow only (as opposed to the bone marrow and bone), to assess the effect of that change on the results. In the simulations, we relied on the following bone marrow parameter ranges: blood flow 0.14 L/min - 0.24 L/min, blood volume 0.16 L - 0.27 L, bone marrow volume 0.85 L - 1.42 L [9]. The simulations with the adjusted parameterisation for the bone marrow compartment resulted in the exact post- to pre-splenectomy SLT T cell localisation and net concentration ratios, and nearly exact model reduction variables. The comparison of the results is shown in Table S.2.

Symbol	Definition	Variable mean (95%CI)	
		Bone marrow and bone parameterisation	Bone marrow parameterisation
κ	Post- to pre-splenectomy cell concentration ratio	1.02 (1 – 1.04)	1.04 (1 – 1.05)
$\frac{\sigma^*\kappa}{\sigma}$	Post- to pre-splenectomy cell concentration ratio: liver-draining lymph nodes	0.97 (0.84 – 1)	0.98 (0.84 – 1)
$\frac{\kappa}{\lambda}$	Post- to pre-splenectomy cell concentration ratio: liver	0.97 (0.84 – 1)	0.99 (0.84 – 1)
λ	Liver pre-splenectomy cell concentration with respect to other organs	1.05 (1 – 1.2)	1.05 (1 – 1.2)
σ	Liver-draining lymph nodes pre-splenectomy cell concentration with respect to other organs	5.58 (1.06 – 30.93)	5.46 (1.06 – 29.97)
σ^*	Liver-draining lymph nodes post-splenectomy cell concentration with respect to other organs	5.18 (1.02 – 28.53)	5.07 (1.02 – 27.57)
$\frac{N^*_{\text{SLT}}}{N_{\text{SLT}}}$	Total SLT post- to pre-splenectomy cell localisation ratio	0.65 (0.12 – 0.97)	0.65 (0.12 – 0.97)
$\frac{C^*_{\text{SLT}}}{C_{\text{SLT}}}$	Net SLT post- to pre-splenectomy cell concentration ratio	0.91 (0.17 – 1.43)	0.91 (0.17 – 1.43)

Table S.2. Comparison of the of post- to pre-splenectomy cell concentration ratios: κ, $\sigma^*\kappa/\sigma$ and κ/λ, as well as λ, σ and σ^*, and the post- to pre-splenectomy total T cell localisation ratio and the net T cell concentration ratio in the SLT simulated for different parameterisation for the skeleton compartment. The results are shown as the variable means and 95%CI for the skeleton compartment parameterisation including the bone marrow and bone contribution, as presented in the Main Text (third column), and the skeleton compartment parameterisation restricted to only bone marrow (fourth column).

References

1. Cazaux, M. *et al.* Single-cell imaging of CAR T cell activity in vivo reveals extensive functional and anatomical heterogeneity. *J. Exp. Medicine* **216**, 1038–1049, DOI: 10.1084/jem.20182375 (2019).

2. Kulkarni, P. *et al.* Imaging Lung Clearance of Radiolabeled Tumor Cells to Study Mice with Normal, Activated or Depleted Natural Killer (NK) Cells. *AIP Conf. Proc.* **680**, DOI: 10.1063/1.1619906 (2003).

3. Ffoulkes-Crabbe, D., Creighton, R., Volgyesi, G., Steward, D. & Nisbet, H. The effect of splenectomy on circulatory adjustments to hypoxaemia in the anaesthetized dog. *Br. J. Anaesth.* **48**, 639–641, DOI: 10.1093/bja/48.7.639 (1976).

4. Lipowsky, H. & Zweifach, B. Network analysis of microcirculation of cat mesentery. *Microvasc. Res.* **7**, 73–83, DOI: 10.1016/0026-2862(74)90038-7 (1974).

5. Pries, A., Secomb, T., Gaehtgens, P. & Gross, J. Blood flow in microvascular networks. Experiments and simulation. *Circ. Res.* **67**, 826–834, DOI: 10.1161/01.res.67.4.826 (1990).

6. Shipley, R., Smith, A., Sweeney, P., Pries, A. & Secomb, T. A hybrid discrete–continuum approach for modelling microcirculatory blood flow. *Math. Medicine Biol. A J. IMA* **37**, 40–57, DOI: 10.1093/imammb/dqz006 (2020).

7. Chilian, W., Layne, S., Klausner, E., Eastham, C. & Marcus, M. Redistribution of coronary microvascular resistance produced by dipyridamole. *The Am. J. Physiol.* **256**, H383–90, DOI: 10.1152/ajpheart.1989.256.2.H383 (1989).

8. Kumar, A., Sharma, P. & Sarin, S. Hepatic venous pressure gradient measurement: time to learn! *Indian J. Gastroenterol. Off. J. Indian Soc. Gastroenterol.* **27**, 74–80 (2008).

9. Snyder, W., Cook, M., Nasset, E., Karhausen, L. & Parry Howells, G. Report on the Task Group on Reference Man. Tech. Rep., ICRP No. 23 (1974).
